# Synergies between synaptic and HCN channel plasticity dictates firing rate homeostasis and mutual information transfer in hippocampal model neuron

**DOI:** 10.3389/fncel.2023.1096823

**Published:** 2023-03-20

**Authors:** Rahul Kumar Rathour, Hanoch Kaphzan

**Affiliations:** Sagol Department of Neurobiology, University of Haifa, Haifa, Israel

**Keywords:** active dendrites, information transfer, voltage-gated ion channels, homeostasis, synaptic plasticity

## Abstract

Homeostasis is a precondition for any physiological system of any living organism. Nonetheless, models of learning and memory that are based on processes of synaptic plasticity are unstable by nature according to Hebbian rules, and it is not fully clear how homeostasis is maintained during these processes. This is where theoretical and computational frameworks can help in gaining a deeper understanding of the various cellular processes that enable homeostasis in the face of plasticity. A previous simplistic single compartmental model with a single synapse showed that maintaining input/output response homeostasis and stable synaptic learning could be enabled by introducing a linear relationship between synaptic plasticity and HCN conductance plasticity. In this study, we aimed to examine whether this approach could be extended to a more morphologically realistic model that entails multiple synapses and gradients of various VGICs. In doing so, we found that a linear relationship between synaptic plasticity and HCN conductance plasticity was able to maintain input/output response homeostasis in our morphologically realistic model, where the slope of the linear relationship was dependent on baseline HCN conductance and synaptic permeability values. An increase in either baseline HCN conductance or synaptic permeability value led to a decrease in the slope of the linear relationship. We further show that in striking contrast to the single compartment model, here linear relationship was insufficient in maintaining stable synaptic learning despite maintaining input/output response homeostasis. Additionally, we showed that homeostasis of input/output response profiles was at the expense of decreasing the mutual information transfer due to the increase in noise entropy, which could not be fully rescued by optimizing the linear relationship between synaptic and HCN conductance plasticity. Finally, we generated a place cell model based on theta oscillations and show that synaptic plasticity disrupts place cell activity. Whereas synaptic plasticity accompanied by HCN conductance plasticity through linear relationship maintains the stability of place cell activity. Our study establishes potential differences between a single compartmental model and a morphologically realistic model.

## Introduction

The instability of synaptic strength during Hebbian plasticity is a major drawback within the frameworks of physiological functioning, computational roles, and synaptic learning. The positive feedback loops incurred during Hebbian plasticity by increase/decrease in AMPA and/or NMDA receptor conductance during repetitive synaptic stimulation could result in complete loss of action potential firing either through a reduction in the synaptic drive during LTD or enhanced synaptic drive during LTP, which eventually could lead to a depolarization-induced block of sodium channels (Guan et al., 2013; Honnuraiah and Narayanan, 2013; Liu and Bean, 2014). Therefore, it is essential to regulate synaptic strengths and responses thereof to provide stability during Hebbian plasticity for maintaining homeostasis of input/output relationship and robust information transfer.

Activity-dependent modifications of rules for synaptic plasticity, defined as metaplasticity, have been postulated to play a key role in the stability during Hebbian plasticity (Bear, 1995; Abraham and Bear, 1996; Abraham and Tate, 1997; Abraham, 2008). Various metaplastic mechanisms have been implicated in providing negative feedback loops for maintaining synaptic stability. Amongst these negative feedback mechanisms are the changes in the subunit composition of NMDA receptors (Philpot et al., 2001), modification in downstream NMDA receptor signaling (Philpot et al., 2003), alteration in calcium buffering (Gold and Bear, 1994), revision of CaMKII levels (Mayford et al., 1995; Bear, 2003), structural plasticity (Matsuzaki et al., 2004; Kalantzis and Shouval, 2009) and presence/plasticity of various voltage-gated ion channels (VGICs) (Narayanan and Johnston, 2010, 2012; Anirudhan and Narayanan, 2015).

Prominent among these is the presence/plasticity of various VGICs in regulating synaptic stability which has received attention in the recent past, since VGICs were shown to express plasticity following synaptic plasticity-inducing protocols (Yasuda et al., 2003; Frick and Johnston, 2005; Magee and Johnston, 2005; Sjostrom et al., 2008; Narayanan and Johnston, 2012). Hyperpolarization-activated cyclic-nucleotide gated (HCN) *h* channel, in particular, has been postulated to play a role in keeping synaptic stability and homeostasis of input/output relationship (Narayanan and Johnston, 2010; Honnuraiah and Narayanan, 2013) owing to the bi-directional plasticity of HCN conductance during synaptic plasticity (Fan et al., 2005; Brager and Johnston, 2007; Narayanan and Johnston, 2007; Campanac et al., 2008). A quantitative modeling framework has established a linear relationship between synaptic and HCN conductance plasticity for maintaining homeostasis of the input/output relationship and robust information transfer (Honnuraiah and Narayanan, 2013). We employed this linear relationship, originally deduced from a single compartmental model having a single synapse, on to a morphologically realistic neuronal model having multiple synapses and expressing gradients of various VGICs for enabling homeostasis of input/output relationship and maintaining robust information transfer. In doing so, we found that the previously derived linear relationship between synaptic plasticity and HCN conductance plasticity in a single compartmental model, having a single synapse for maintaining input/output response homeostasis, could be extended to multi-compartmental model having multiple synapses and gradients of various ion-channels, where the optimal slope of the linear relationship between synaptic and HCN conductance plasticity is heavily dependent upon synaptic permeability values and baseline HCN conductance levels. We also found that homeostasis of the input/output response profile does not necessarily translate to robust information transfer. Finally, using a Gaussian-modulated input pattern, we show that HCN conductance plasticity along with synaptic plasticity could provide stability to place cell firing within the place field. Our study provides useful insights in terms of homeostasis, and interdependence between input/output relationship and information transfer, and thereby underscores the importance of crosstalk between synaptic and intrinsic plasticity in regulating learning and homeostasis in single neurons and their networks.

## Materials and methods

A morphologically realistic, 3D reconstructed, hippocampal CA1 pyramidal neuron (*n123*), obtained from Neuromorpho.org (Ascoli et al., 2007) was used as the substrate for all simulations. Morphology and modeling parameters of passive membrane properties and voltage-gated ion channels (VGICs) were the same as those used in previous studies (Rathour and Narayanan, 2014; Rathour and Kaphzan, 2022) and are detailed below.

### Passive membrane properties

Passive membrane parameters were set such that the model neuron was able to capture experimental statistics of various measurements (Hoffman et al., 1997; Magee, 1998; Migliore et al., 1999; Narayanan and Johnston, 2007, 2008). Explicitly, specific membrane capacitance (*C*_m_) was set at 1 μF/cm^2^ across the entire morphology. Specific membrane resistivity (*R*_m_) and intracellular resistivity (*R*_a_) were distributed non-uniformly and varied along the somato-apical trunk as functions of the radial distance of the compartment from the soma (*x*) using the following formulation:

(1) $$\begin{array}{l} R_{\text{m}}\left(x\right)=R_{\text{m}}-\text{max}+\frac{\left(R_{\text{m}}-\text{min}-R_{\text{m}}-\text{max}\right)}{1+\exp\left(\left(R_{\text{m}}-d-x\right)/R_{\text{m}}-k\right)} \end{array}$$

(2) $$\begin{array}{l} R_{\text{a}}\left(x\right)=R_{\text{a}}-\text{max}+\frac{\left(R_{\text{a}}-\text{min}-R_{\text{a}}-\text{max}\right)}{1+\exp\left(\left(R_{\text{a}}-d-x\right)/R_{\text{a}}-k\right)} \end{array}$$

where *R*_m_ – max = 125 kΩ/cm^2^ and *R*_a_ – max = 120 Ω/cm were default values at the soma, and *R*_m_ – min = 85 kΩ/cm^2^ and *R*_a_ – min = 70 Ω/cm were values assigned to the terminal end of the apical trunk (which was ~425 μm distance from the soma for the reconstruction under consideration). The other default values were: *R*_m_ – *d* = *R*_a_ – *d* = 300 μm, *R*_m_ – *k* = R_a_ – *k* = 50 μm; *R*_a_ – *k* = 14 μm. The basal dendrites and the axonal compartments had somatic *R*_m_ and *R*_a_. Model neuron with these distributions of passive membrane properties was compartmentalized using *d*_λ_ rule (Carnevale and Hines, 2006) to ensure that each compartment was smaller than 0.1λ_100_, where λ_100_ was the space constant computed at 100 Hz. This produced a total of 809 compartments in the model neuron.

### Voltage-gated ion channels kinetics and distribution

The model neuron used expressed five conductance-based voltage-gated ion channels (VGICs): Na^+^, *A*-type K^+^ (KA), delayed rectifier K^+^ (KDR), *T*-type Ca^++^ (CaT), and hyperpolarization-activated cyclic-nucleotide gated (HCN) *h* channels. Na^+^, KDR, and KA channels were modeled based on previous kinetic schemes (Migliore et al., 1999), and *h* channels were modeled as in Poolos et al. (2002). *T*-type Ca^++^ channels kinetics was taken from Shah et al. (2008). Na^+^, K^+^, and *h* channels models were based upon Hodgkin-Huxley formalism and had reversal potentials 55, −90, and −30 mV respectively. The CaT current was modeled using the Goldman-Hodgkin-Katz (GHK) formulation with the default values of external and internal Ca^++^ concentrations set at 2 mM and 100 nM, respectively. The Densities of Na and KDR conductances were kept uniform across the neuronal arbor, whereas the densities of h, CaT, and KA channel conductances increased on the apical side with an increase in distance from the soma (Magee and Johnston, 1995; Hoffman et al., 1997; Magee, 1998). The basal dendritic compartments had somatic conductance values.

**Figure 1:**
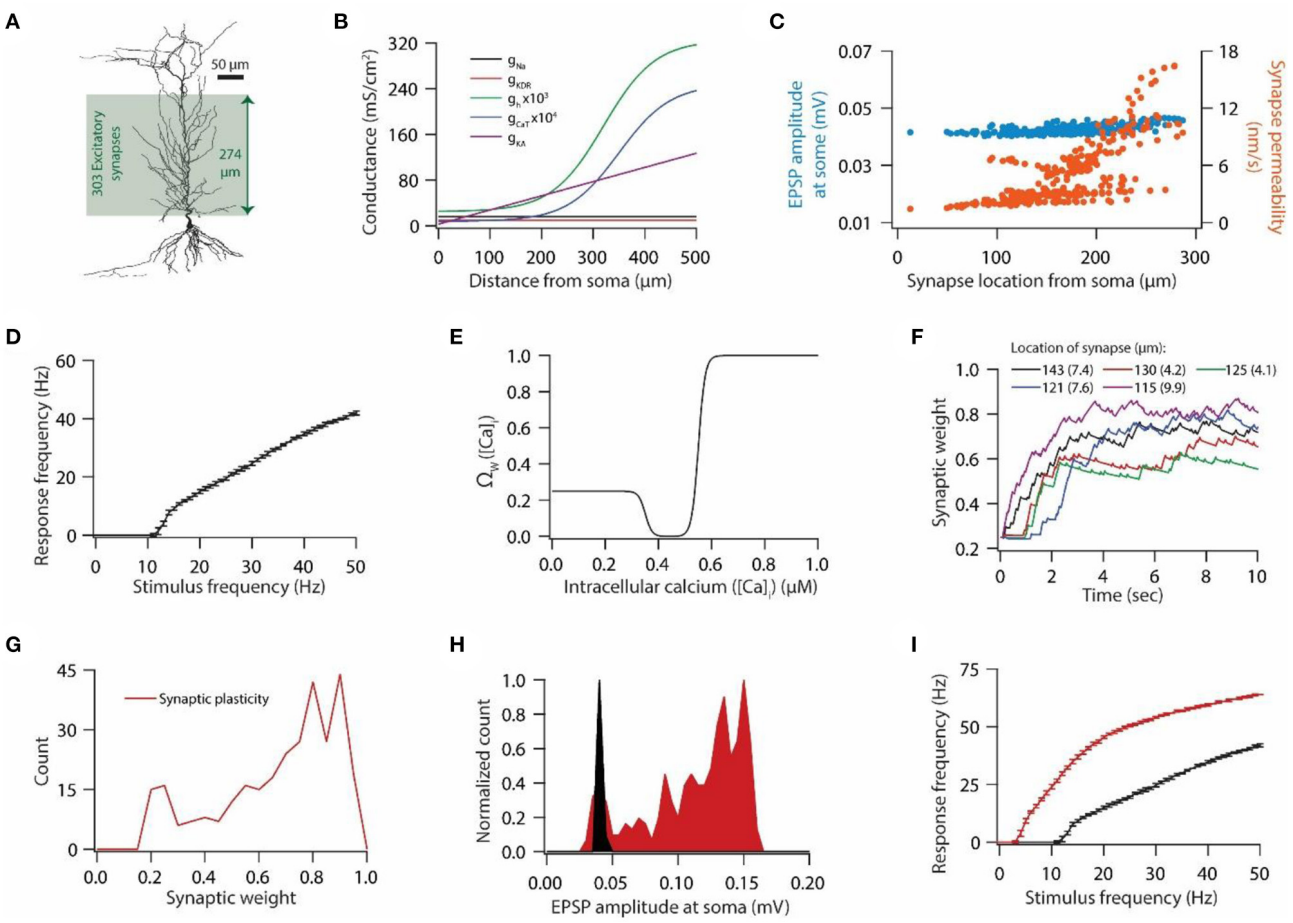
Synaptic plasticity disrupts input/output response homeostasis. **(A)** 3D reconstructed morphology of CA1 pyramidal neuron used in this study. **(B)** Distribution of various voltage-gated ion channels along the apical side of dendritic arbor. **(C)** Somatic EPSP amplitudes (cyan) of 303 synapses located across the apical dendritic arbor [green region in **(A)**] and their corresponding maximum permeability values (orange). **(D)** Input/output relationship of the model neuron under the baseline condition. All synapses were stimulated at a given frequency with stimulation timings drawn from the independent Poisson distribution. Stimulation of a synapse at a given frequency was repeated 10 times. Data is presented as mean ± SD. **(E)** Ω-function used in this study to update synaptic weight as a function of intracellular Ca^++^ concentration. **(F)** Example traces for evolution of synaptic weights recorded at various locations. Each of the 303 synapses was activated at a given frequency, assigned from a uniform distribution of 4–12 Hz range. The stimulation timings of each synapse were Poisson distributed. Number within parenthesis against distance denotes the stimulus frequency. **(G)** Distribution of final synaptic weights across all 303 synapses. Bin size 0.05. **(H)** Distribution of somatic EPSP amplitudes of 303 synapses under the baseline condition (black) and after synaptic plasticity (red). Bin size 5 μV. **(I)** Input/output response profiles of the model neuron under baseline condition (black) and after synaptic plasticity (red). Data is presented as mean ± SD.

**Figure 2:**
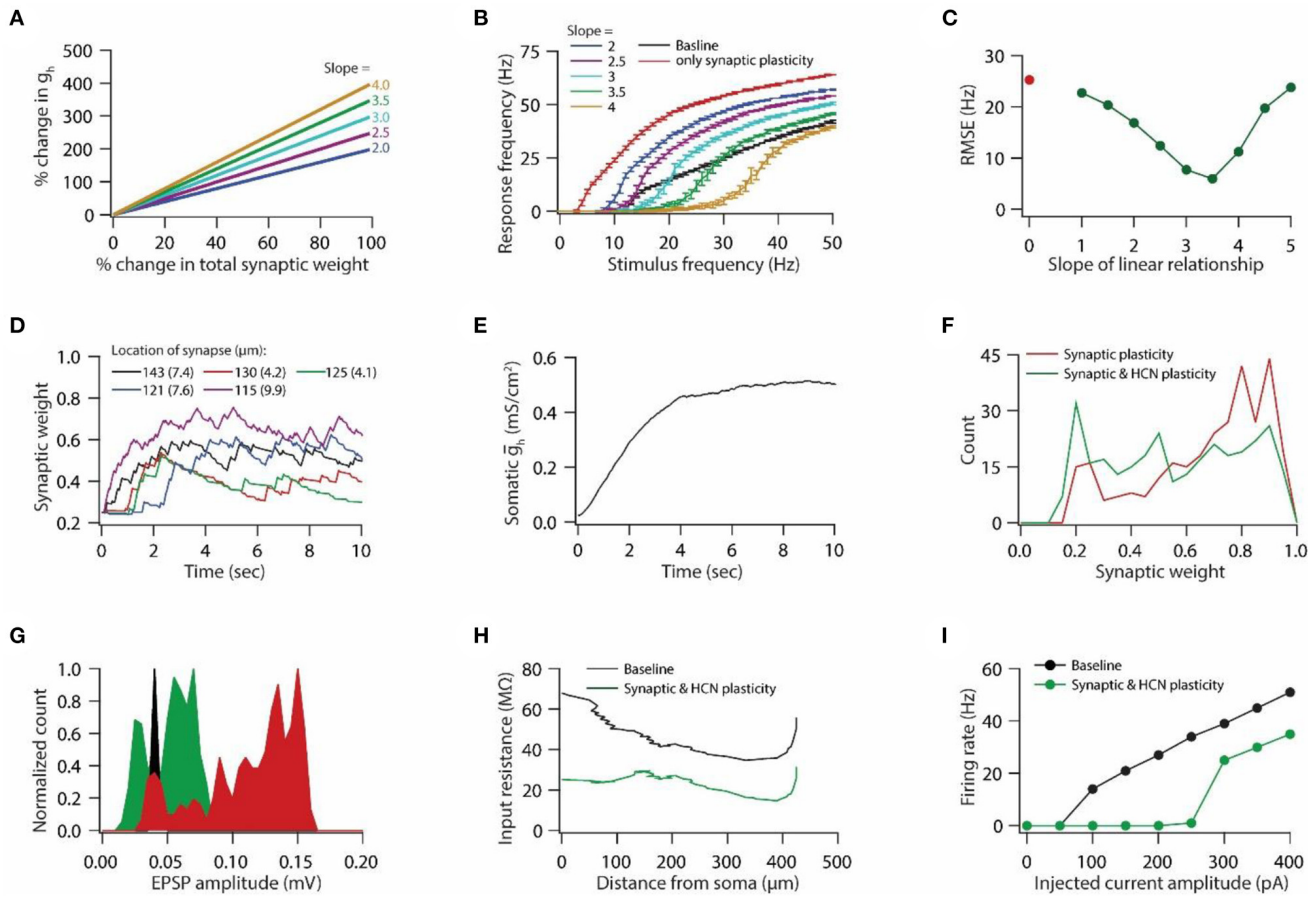
Synaptic plasticity accompanied with HCN conductance plasticity enables homeostasis of input/output response profiles. **(A)** Example graph of linear relationship between percentage change in total synaptic weight and percentage change in HCN conductance for various slopes. **(B)** Input/output response profiles of the model neuron under baseline condition (black), after only synaptic plasticity (red) and after synaptic and HCN conductance plasticity for various slopes of the linear relationship **(A)**. Data is presented as mean ± SD. **(C)** Root mean squared error (RMSE) between baseline input/output response profile and response profile obtained after synaptic and HCN conductance plasticity (green trace) as a function of slope of the linear relationship **(A)**. Red dot denotes RMSE between baseline input/output response profile and response profile obtained after only synaptic plasticity (Figure 1I). **(D)** Example traces for evolution of synaptic weights recorded at various locations (same as in Figure 1F). Number within parenthesis against distance denotes stimulus frequency in Hz. **(E)** Example trace of evolution of somatic HCN conductance during synaptic and HCN conductance plasticity for optimal slope (3.5) to yield RMSE minimization **(C)**. **(F)** Distribution of final synaptic weights across all 303 synapses after only synaptic plasticity (red) and after synaptic and HCN conductance plasticity (green). Bin size 0.05. **(G)** Histogram of somatic EPSP amplitudes of 303 synapses under baseline condition (black), after synaptic plasticity (red) and after synaptic and HCN conductance plasticity (green). **(H)** Input resistance along the neuronal trunk computed under baseline condition (black) and after synaptic and HCN conductance plasticity (green). **(I)** Intrinsic firing rate profile at soma under baseline condition (black) and after synaptic and HCN conductance plasticity (green).

**Figure 3:**
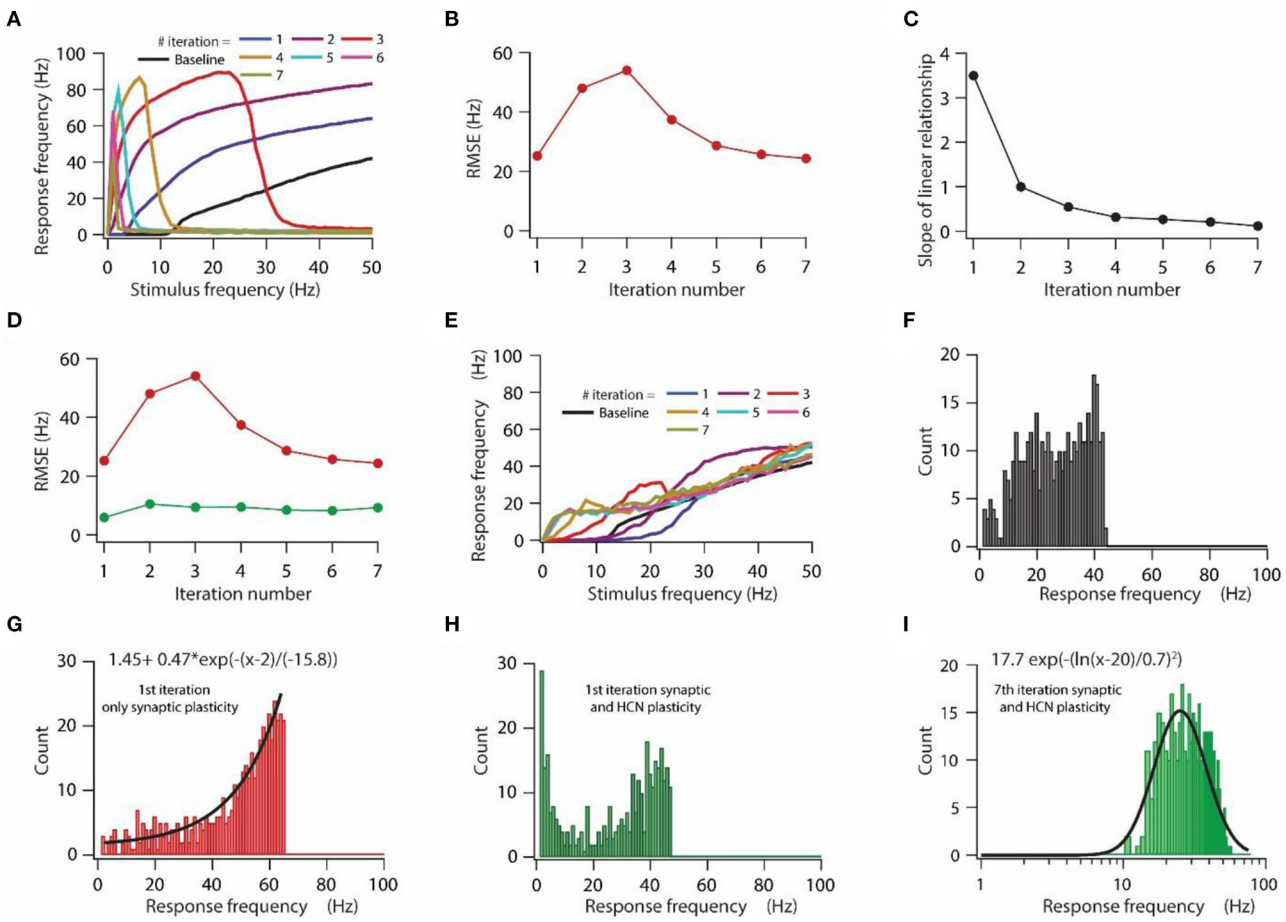
HCN conductance plasticity along with synaptic plasticity maintains homeostasis of input/output response profile during repeated synaptic stimulation. **(A)** Input/output response profiles of the model neuron under baseline condition (black) and after various iteration of synaptic stimulation. Note the complete cessation of response firing rate after 5th, 6th, and 7th iteration. Stimulation patterns for inducing synaptic plasticity of all synapses were same across all iterations. **(B)** RMSE between baseline input/output response profile and response profile obtained after repeated synaptic plasticity. **(C)** Optimal slope of the liner relationship between synaptic and HCN conductance plasticity as a function of the number of iterations of synaptic stimulation for minimizing RMSE. **(D)** RMSE between baseline input/output response profile and response profile obtained after synaptic and HCN conductance plasticity (green trace) as a function of the number of iterations of synaptic stimulation. Red trace same as in **(B)**. **(E)** Input/output response profile of the model neuron under baseline condition (black) and after various iterations of synaptic stimulation under the condition of synaptic and HCN conductance plasticity. **(F–I)** Distributions of response frequencies computed across all stimulus frequencies and trials under baseline condition **(F)**, 1st iteration of only synaptic plasticity **(G)**, 1st iteration of synaptic and HCN conductance plasticity **(H)** and 7th iteration of synaptic and HCN conductance plasticity **(I)**. Bin size 1 Hz.

**Figure 4:**
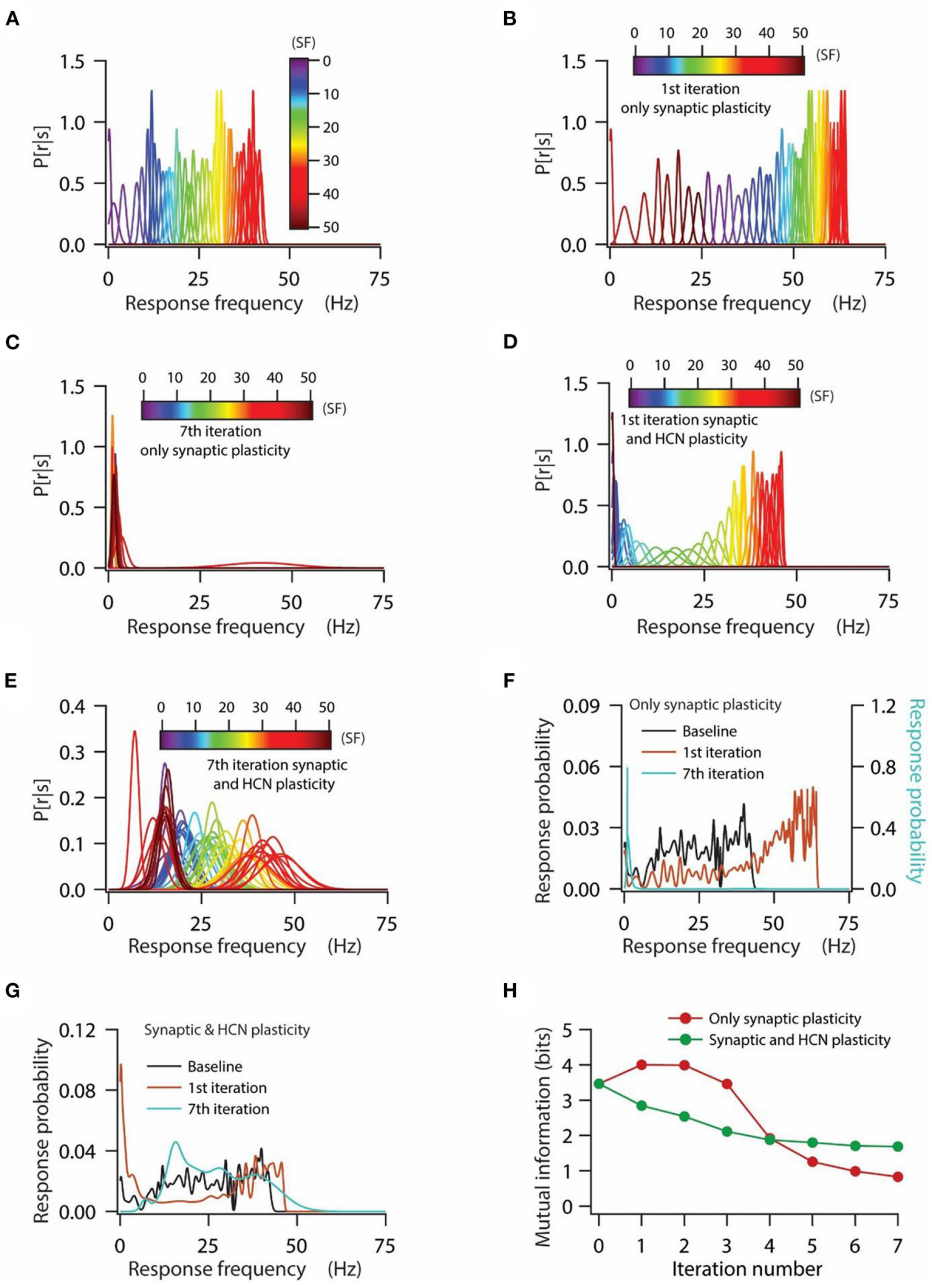
HCN conductance plasticity along with synaptic plasticity maintains homeostasis of input/output response profiles at the expense of mutual information transfer. **(A)** Distribution of probability of response firing rates for various stimulus frequencies (*p*[r|s]) under baseline condition. **(B, C)** Distribution of probability of response firing rates for various stimulus frequencies (*p*[r|s]) after 1st iteration **(B)** and 7th iteration **(C)** of only synaptic plasticity. **(D, E)** Distribution of probability of response firing rates for various stimulus frequencies (*p*[r|s]) after 1st iteration **(D)** and 7th iteration **(E)** of synaptic plasticity along with HCN conductance plasticity. **(F)** Probability distribution of various response firing rates under baseline condition (black) and after 1st iteration (brown) and 7th iteration (cyan) of only synaptic plasticity. **(G)** Probability distribution of various response firing rates under baseline condition (black) and after 1st iteration (brown) and 7th iteration (cyan) of synaptic plasticity along with HCN conductance plasticity. **(H)** Mutual information as a function of repeated iterations with introducing only synaptic plasticity (red) and repeated iterations with introducing synaptic and HCN conductance plasticity. Zero iteration denotes baseline condition.

**Figure 5:**
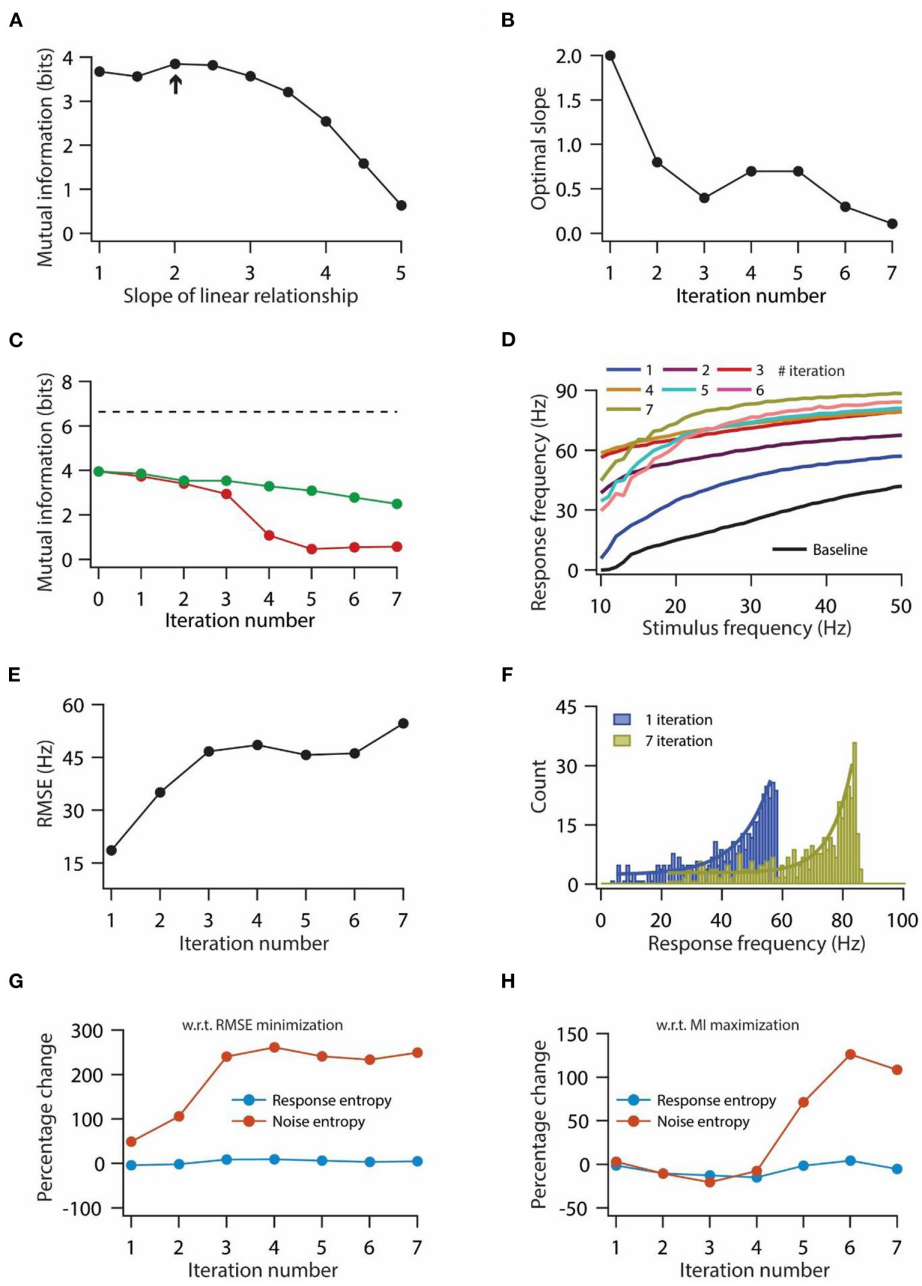
Optimization of slope for information maximization also leads to a decrease in information transfer during repeated synaptic stimulation. **(A)** Mutual information as a function of slope of the linear relationship between synaptic and HCN conductance plasticity. Arrow indicates the optimal slope. **(B)** Optimal slope of the liner relationship between synaptic and HCN conductance plasticity as a function of the number of iterations of synaptic stimulation for maximizing mutual information transfer. **(C)** Mutual information transfer as a function of the number of iterations of synaptic stimulation under the condition of optimal slope between synaptic and HCN conductance plasticity for maximizing information transfer **(B)** (green trace) and only synaptic plasticity (red). Dashed line represents theoretically maximum information transfer computed under the assumption of zero noise entropy and uniform distribution of response probability within the range of 1–100 Hz of response frequencies. **(D)** Input/output response profiles of model neuron under baseline condition (black) and after various iterations of synaptic stimulation under the condition of optimal slope between synaptic and HCN conductance plasticity for maximizing information transfer **(B)**. **(E)** RMSE between baseline input/output response profile and response profile obtained after synaptic and HCN conductance plasticity, as a function of the number of iterations of synaptic stimulation under the condition of optimal slope between synaptic and HCN conductance plasticity for maximizing information transfer **(B)**. **(F)** Distributions of response frequencies computed across all stimulus frequencies and trials for 1st and 7th iteration of synaptic and HCN conductance plasticity. Bin size 1 Hz. **(G, H)** Percentage change in response and noise entropy as a function of the number of iterations of synaptic stimulation under the condition of RMSE minimization **(G)** and maximization of mutual information transfer **(H)**.

For simulations involving Poisson-modulated synaptic inputs (Figures 1–5), uniformly distributed Na and KDR conductances were set at 16 and 10 mS/cm^2^, respectively. Na conductance was five-fold higher in the axon initial segment compared to the somatic counterpart (Fleidervish et al., 2010), and the rest of the axon was treated as passive. To account for the slow inactivation of dendritic Na^+^ channels, an additional inactivation gating variable was included for dendritic Na^+^ channels (Migliore et al., 1999). KA conductance was set as a linearly increasing gradient as a function of radial distance from the soma, *x* (Hoffman et al., 1997), using the following formulation:


(3) $$\begin{array}{l} {\bar{g}}_{\text{KA}}\left(x\right)=A-g_{\text{B}}\;\left(1+A-Fx/100\right) \end{array}$$

where somatic ${\bar{g}}_{\text{KA}}$ was 3.1 mS/cm^2^, and *A* – *F* (=8) quantified the slope of this linear gradient. In order to incorporate incremental observations related to differences in half-maximal activation voltage (*V*_1/2_) between the proximal and the distal KA channels in CA1 pyramidal cells (Hoffman et al., 1997), two distinct models of KA channels were adopted. A proximal model was used for compartments with radial distances < 100 μm from the soma, and beyond that point, a distal *A*-type K^+^ conductance model was used.

The increase in maximal *h* conductance along the somato-apical axis as a function of radial distance from the soma, *x*, was modeled using the following formulation:

(4) $$\begin{array}{l} {\bar{g}}_{\text{h}}\left(x\right)=h-g_{\text{B}}\;\left(1+\frac{h-F}{1+\exp\left(\left(h-d-x\right)/h-k\right)}\right) \end{array}$$

where *h* – *g*_B_ denotes maximal *h* conductance at the soma, set to be 25 μS/cm^2^, and *h* – *F* (=12) formed fold increase along the somato-apical axis. Half-maximal distance of ${\bar{g}}_{h}$ increase, *h* – *d* was 320 μm, and the parameter quantifying the slope, *h* – *k* was 50 μm. To accommodate the experimental observations regarding changes in *V*_1/2_ of the activation of *h* conductance at various locations along the somato-apical trunk (Magee, 1998), the half-maximal activation voltage for *h* channels was −82 mV for *x* ≤ 100 μm, linearly varied from −82 to −90 mV for 100 μm ≤ *x* ≤ 300 μm, and −90 mV for *x* > 300 μm.

The CaT conductance gradient was modeled as a sigmoidal increase with increasing radial distance from the soma, *x*:

(5) $$\begin{array}{l} {\bar{g}}_{\text{CaT}}\left(x\right)=T-g_{\text{B}}\;\left(1+\frac{T-F}{1+\exp\left(\left(T-d-x\right)/T-k\right)}\right) \end{array}$$

where *T* – *g*_B_ denotes maximal CaT conductance at the soma, set to be 80 μS/cm^2^, and *T* – *F* (=30) formed fold increase along the somato-apical axis. Half-maximal distance of ${\bar{g}}_{\text{CaT}}$ increase, *T* – *d* was 350 μm, and the parameter quantifying the slope, *T* – *k* was 50 μm. These parametric constrains accounted for the experimental constraints on the coexistence of the six functional maps along the same somato-apical trunk (Rathour and Narayanan, 2014).

**Figure 6:**
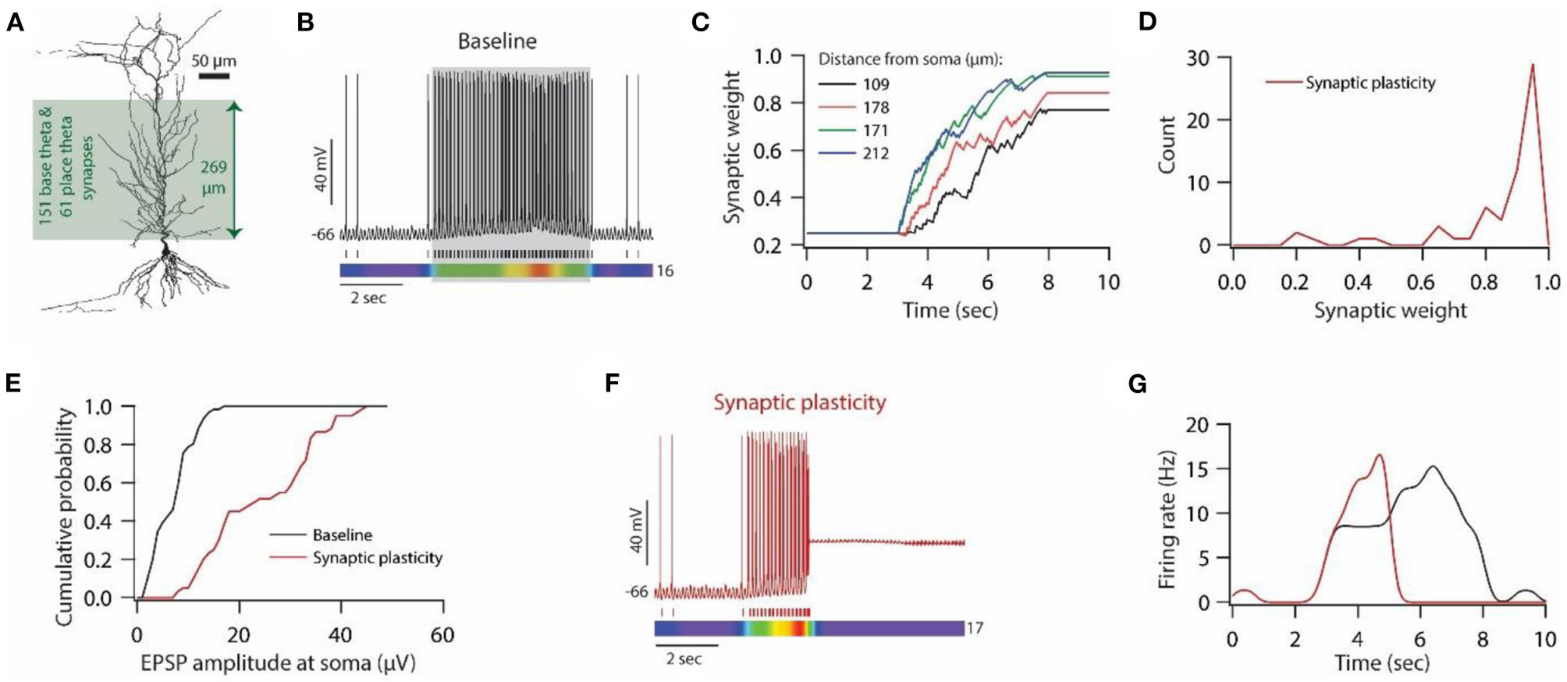
Synaptic plasticity disrupts place field activity in the model neuron. **(A)** 3D reconstructed morphology of CA1 pyramidal neuron used for simulating place field activity. **(B)** *Top*; Voltage trace recorded during Gaussian- modulated stimulation of base and place theta synapses along with asymmetric depolarizing ramp current injection at the soma under baseline condition. *Middle*; Spike timings. *Bottom*; kymograph of firing rate. Number at the right denotes the maximum firing rate in Hz. **(C)** Example traces for evolution of synaptic weights recorded at various locations. **(D)** Distribution of final synaptic weights across all place theta synapses. Bin size 0.05. **(E)** Cumulative probability distribution of somatic EPSP amplitudes of place theta synapses under the baseline condition (black) and after synaptic plasticity (red). Bin size 5 μV. **(F)** *Top*; Voltage trace recorded during Gaussian-modulated stimulation of base and place theta synapses along with asymmetric depolarizing ramp current injection at the soma under synaptic plasticity condition. *Middle*; Spike timings. *Bottom*; kymograph of firing rate. Number at the right denotes the maximum firing rate in Hz. **(G)** Place field activity under baseline condition (black trace) and after synaptic plasticity (red trace).

**Figure 7:**
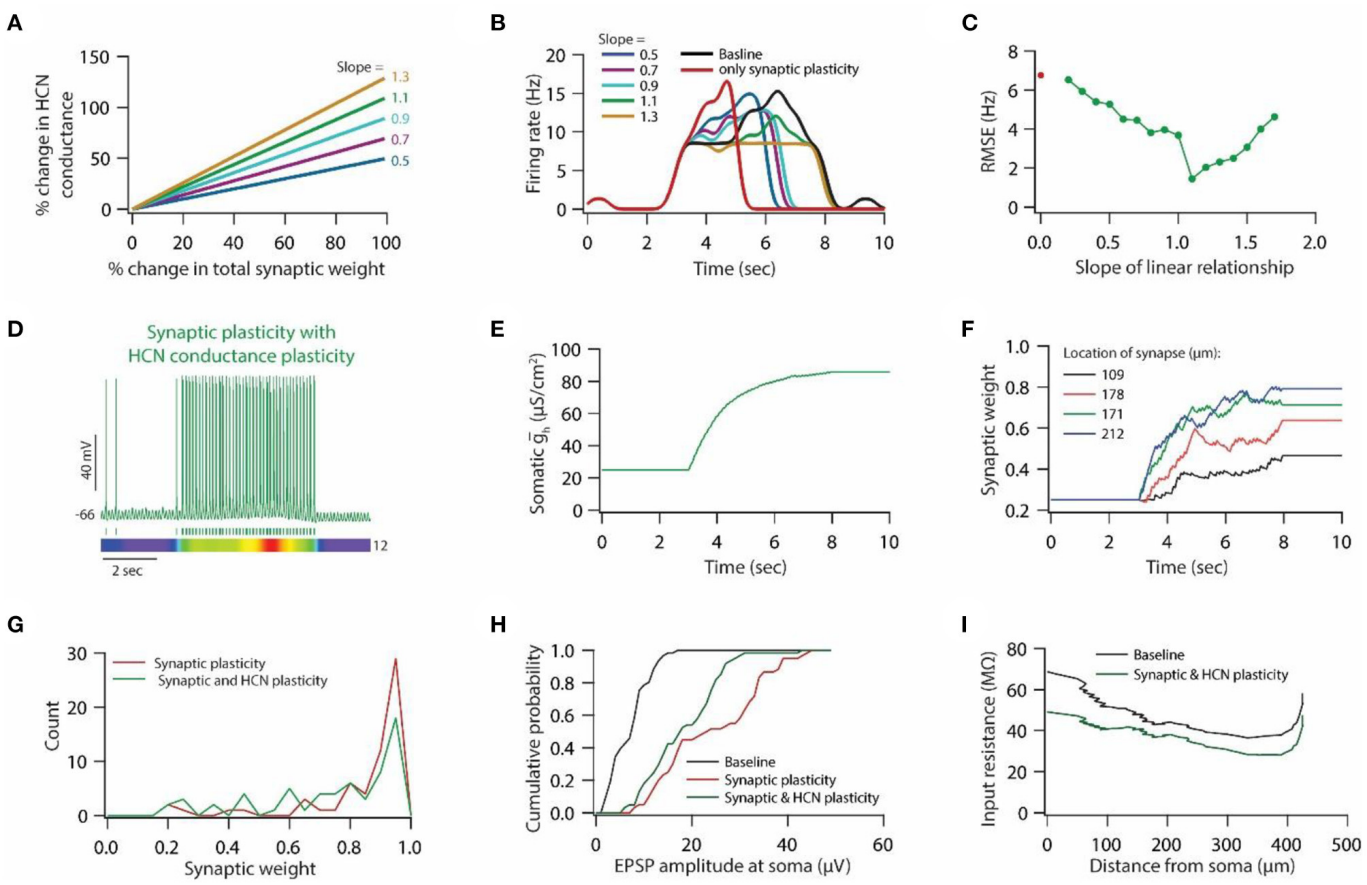
HCN conductance plasticity along with synaptic plasticity restores place field activity. **(A)** Example graph of linear relationship between percentage change in total synaptic weight and percentage change in HCN conductance for various slopes. **(B)** Place field profiles of model neurons under baseline condition (black), after only synaptic plasticity (red) and after synaptic and HCN conductance plasticity for various slopes of the linear relationship. **(C)** RMSE between baseline place field profile and place field profile obtained after synaptic and HCN conductance plasticity (green trace) as a function of slope of the liner relationship. Note, optimal slope is at 1.1. Red dot denotes RMSE between baseline place field profile and place field profile obtained after only synaptic plasticity (Figure 6G). **(D)** *Top*; Voltage trace recorded during Gaussian-modulated stimulation of base and place theta synapses along with asymmetric depolarizing ramp current injection at the soma under the conditions of synaptic and HCN conductance plasticity for slope 1.1 (optimal slope) of linear relationship. *Middle*; Spike timings. *Bottom*; kymograph of firing rate. Number at the right denotes maximum firing rate in Hz. **(E)** Evolution of somatic HCN conductance during synaptic and HCN conductance plasticity for slope 1.1 (optimal slope) of linear relationship. **(F)** Example traces for evolution of synaptic weights recorded at various locations (same as in Figure 6C). **(G)** Distribution of synaptic weights across all place theta synapses after synaptic plasticity (red) and after synaptic and HCN conductance plasticity (green). Bin size 0.05. **(H)** Cumulative probability distribution of somatic EPSP amplitudes of place theta synapses under baseline condition (black), after synaptic plasticity (red) and after synaptic and HCN conductance plasticity (green). Bin size 5 μV. **(I)** Input resistance along the neuronal trunk computed under baseline condition (black) and after synaptic and HCN conductance plasticity (green).

For simulations involving Gaussian-modulated synaptic inputs (Figures 6, 7), the parameters used for kinetics, distributions, and maximal conductances of KA, CaT, and HCN channels were the same as aforementioned, whereas maximal Na and KDR conductances were set at 15.4 and 2 mS/cm^2^, respectively. After changing these conductances, the model neuron was able to satisfy experimental constraints on the coexistence of the six functional maps along the same somato-apical trunk.

### Synapse model and distribution

A synapse was modeled as a co-localization of AMPA and NMDA receptor currents as described previously (Narayanan and Johnston, 2010; Honnuraiah and Narayanan, 2013). A spike generator was used to feed inputs to the synapses at predetermined required frequencies. The default value of the ratio of NMDA:AMPA permeability was set at 1.5. Both receptor currents were modeled based on GHK formulation. The current through NMDA receptors was a combination of Na^+^, K^+^, and Ca^++^, and their voltage and time dependence were described by the following equations:

(6) $$\begin{array}{l} I_{\text{NMDA}}\left(v,t\right)=I_{\text{NMDA}}^{\text{Na}}\left(v,t\right)+I_{\text{NMDA}}^{\text{K}}\left(v,t\right)+I_{\text{NMDA}}^{\text{Ca}}\left(v,t\right) \end{array}$$

where

(7) $$\begin{array}{l} I_{\text{NMDA}}^{\text{Na}}\left(v,t\right)={\bar{P}}_{\text{NMDA}}P_{\text{Na}}s\left(t\right)MgB\left(v\right)\frac{vF^{\text{2}}}{RT}\left\{\frac{{\left[Na\right]}_{\text{i}}-{\left[Na\right]}_{\text{o}}\exp\left(-\frac{vF}{RT}\right)}{1-\exp\left(-\frac{vF}{RT}\right)}\right\} \end{array}$$

(8) $$\begin{array}{l} I_{\text{NMDA}}^{\text{K}}\left(v,t\right)={\bar{P}}_{\text{NMDA}}P_{\text{K}}s\left(t\right)MgB\left(v\right)\frac{vF^{\text{2}}}{RT}\left\{\frac{{\left[K\right]}_{\text{i}}-{\left[K\right]}_{\text{o}}\exp\left(-\frac{vF}{RT}\right)}{1-\exp\left(-\frac{vF}{RT}\right)}\right\} \end{array}$$

(9) $$\begin{array}{l} I_{\text{NMDA}}^{\text{Ca}}\left(v,t\right)={\bar{P}}_{\text{NMDA}}P_{\text{Ca}}s\left(t\right)MgB\left(v\right)\frac{4vF^{\text{2}}}{RT}\left\{\frac{{\left[Ca\right]}_{\text{i}}-{\left[Ca\right]}_{\text{o}}\exp\left(-\frac{2vF}{RT}\right)}{1-\exp\left(-\frac{2vF}{RT}\right)}\right\} \end{array}$$

where *F* is Faraday's constant, *R* is the gas constant and *T* is the temperature in Kelvin. ${\bar{P}}_{\text{NMDA}}$ is the maximum permeability of NMDA receptor and the default ratio of values of P_Ca_, P_Na_, and P_K_ was set to be 10.6:1:1, respectively, owing to experimental observations (Mayer and Westbrook, 1987; Canavier, 1999). The external and internal concentrations of the various ions were set as follows (in mM): [Na]_o_ = 140, [Na]_i_ = 18, [K]_o_ = 5, [K]_i_ = 140, [Ca]_o_ = 2, [Ca]_i_ = 100 × 10^−6^. This resulted in equilibrium potentials for sodium and potassium ions +55 and −90 mV, respectively. *MgB*(*v*) and *s*(*t*) denote magnesium dependence and temporal evolution of NMDA current, respectively, and were defined as follows (Jahr and Stevens, 1990a,b):

(10) $$\begin{array}{l} MgB\left(v\right)={\left\{1+\frac{{\left[Mg\right]}_{\text{o}}exp\left(-0.062v\right)}{3.57}\right\}}^{-1} \end{array}$$

where [Mg]_o_ denotes extracellular magnesium concentration and was set to 2 mM.

(11) $$\begin{array}{l} s\left(t\right)=a\left[exp\left(-\frac{t}{\tau_{\text{d}}}\right)-exp\left(-\frac{t}{\tau_{\text{r}}}\right)\right] \end{array}$$

where *a* is the normalization constant to insure that 0 ≤ *s*(*t*) ≤ 1. τ_r_ and τ_d_ denote the rise and decay time constants of NMDA receptor-mediated current, respectively, and were set to be 5 and 50 ms, respectively.

The evolution of intracellular calcium, consequent to entry from NMDA receptors and *T*-type Ca^++^ channels, was modeled as described previously (Poirazi et al., 2003; Narayanan and Johnston, 2010):

(12) $$\begin{array}{l} \frac{d{\left[Ca\right]}_{i}}{dt}=-\frac{10,000I_{\text{NMDA}}^{Ca}}{3.6.dpt.F}+\frac{{\left[Ca\right]}_{\infty }-{\left[Ca\right]}_{i}}{\tau_{\text{Ca}}} \end{array}$$

where τ_Ca_ = 30 ms is the calcium decay time constant, *dpt* = 0.1 μm is the depth of the shell and [Ca]_∞_ = 10^−4^ mM is the steady-state value of [Ca]_i_.

The current through AMPA receptors was mediated by the combination of Na^+^ and K^+^ currents and was defined as follows:

(13) $$\begin{array}{l} I_{\text{AMPA}}\left(v,t\right)=I_{\text{AMPA}}^{\text{Na}}\left(v,t\right)+I_{\text{AMPA}}^{\text{K}}\left(v,t\right) \end{array}$$

where

(14) $$\begin{array}{l} I_{\text{AMPA}}^{\text{Na}}\left(v,t\right)={\bar{P}}_{\text{AMPA}}wP_{\text{Na}}s\left(t\right)\frac{vF^{\text{2}}}{RT}\left\{\frac{{\left[Na\right]}_{\text{i}}-{\left[Na\right]}_{\text{o}}\exp\left(-\frac{vF}{RT}\right)}{1-\exp\left(-\frac{vF}{RT}\right)}\right\} \end{array}$$

(15) $$\begin{array}{l} I_{\text{AMPA}}^{\text{K}}\left(v,t\right)={\bar{P}}_{\text{AMPA}}wP_{\text{K}}s\left(t\right)\frac{vF^{\text{2}}}{RT}\left\{\frac{{\left[K\right]}_{\text{i}}-{\left[K\right]}_{\text{o}}\exp\left(-\frac{vF}{RT}\right)}{1-\exp\left(-\frac{vF}{RT}\right)}\right\} \end{array}$$

where ${\bar{P}}_{\text{AMPA}}$ is the maximum permeability of AMPA receptors. The default ratio of values of P_Na_ and P_K_ was set to be 1:1 owing to experimental observations (Dingledine et al., 1999). *s*(*t*) denotes the temporal evolution of AMPA current and was modeled as in equation (11) with τ_r_ and τ_d_ set to be 2 and 10 ms, respectively. *w* is the weight parameter that undergoes activity-dependent update (see section Synaptic weight update mechanism).

Synapses were distributed across the apical dendritic arbor in the range of 12.5 to 286.7 μm away from the soma. Within this distance range, each compartment was assigned a single synapse.

### Synaptic weight update mechanism

The synaptic weight parameter, *w*, associated with the specific synapse of the given compartment was updated based on the intracellular calcium concentration of the given compartment. This dependence of synaptic weight parameter, *w*, on intracellular calcium concentration was defined by the following equation, based upon the calcium control hypothesis (Shouval et al., 2002):

(16) $$\begin{array}{l} \frac{dw}{dt}=\eta \left({\left[Ca\right]}_{\text{i}}\right)\left[\Omega \left({\left[Ca\right]}_{\text{i}}\right)-w\right] \end{array}$$

where η([Ca]_i_) is the calcium-dependent learning rate, and was dependent upon learning time constant τ([Ca]_i_) as follows:

(17) $$\begin{array}{l} \eta \left({\left[Ca\right]}_{\text{i}}\right)=\frac{1}{\tau \left({\left[Ca\right]}_{\text{i}}\right)} \end{array}$$

where τ([Ca]_i_) was defined as:

(18) $$\begin{array}{l} \tau \left({\left[Ca\right]}_{\text{i}}\right)=P_{1}+\frac{P_{2}}{P_{3}+{\left[Ca\right]}_{\text{i}}^{{\text{P}}_{\text{4}}}} \end{array}$$

with *P*_1_ = 1 s, *P*_2_ = 0.1 s, *P*_3_ = *P*_2_ × 10^−4^ and *P*_4_ = 3. The values of these parameters warrant that when [Ca] ≈ 0, τ([Ca]_i_) ≈ 3 h.

Ω([Ca]_i_) has the following form:

(19) $$\begin{array}{l} \Omega \left({\left[Ca\right]}_{\text{i}}\right)=0.25+\frac{1}{1+\exp\left\{-\beta_{2}\left({\left[Ca\right]}_{\text{i}}-\alpha_{2}\right)\right\}}\\ \;-0.25\frac{1}{1+\exp\left\{-\beta_{1}\left({\left[Ca\right]}_{\text{i}}-\alpha_{1}\right)\right\}} \end{array}$$

with α_1_ = 0.35, α_2_ = 0.55, β_1_ = 80 and β_2_ = 80. The default initial value of *w*, *w*_init_, was set at 0.25.

### HCN conductance update rule

Owing to the previously derived linear relationship between synaptic plasticity and HCN conductance plasticity in order to maintain firing rate homeostasis, and given the experimental observation that localized induction of LTP results in a widespread increase in HCN conductance (Narayanan and Johnston, 2007, 2008), we formulated the dependence of HCN conductance on synaptic weights as follows:

(20) $$\begin{array}{l} g_{h}^{t+\Delta t}=g_{h}^{t}+\left(g_{h}^{t}\cdot \Delta W\cdot Slope\right) \end{array}$$

where $g_{h}^{t}$ and $g_{h}^{t+\Delta t}$ is the maximal somatic HCN conductance (*h*-*g*_B_, Equation 4) at time *t* and *t*+Δ*t*. *Slope* is the slope of the linear relationship and Δ*W* is the percentage change in total synaptic weight across all synapses and was computed as follows:

(21) $$\begin{array}{l} \Delta W=\frac{\overset{i}{\underset{0}{\sum }}w_{i}^{t+\Delta t}-\overset{i}{\underset{0}{\sum }}w_{i}^{t}}{\overset{i}{\underset{0}{\sum }}w_{i}^{t}} \end{array}$$

where $w_{i}^{t}$ and $w_{i}^{t+\Delta t}$ are weights of *i*th synapse at time *t* and *t* + Δ*t*.

### Measurements

The input/output relationship of the model neuron was determined by stimulating synapses at various frequencies. For any given input frequency, all synapses were stimulated simultaneously using independent Poisson distributed input timings and this was repeated 10 times for a given stimulus frequency. Each trial ran for 1 s and the number of action potentials fired was taken as the response frequency. The firing rate in response to direct current pulse injection at soma was determined by injecting currents at various amplitudes for 1 s, and the number of action potentials fired was taken as the firing rate.

EPSP amplitude was computed by activating a given synapse at a given location, and the corresponding potential was recorded at the soma. The difference between baseline potential and peak EPSP response was taken as EPSP amplitude. For computing synaptically driven input/output response profile and EPSP amplitude, only AMPA receptor type conductance was used (Magee and Cook, 2000).

The input resistance of the model neuron at various locations along the neuronal trunk was computed by injecting a current pulse of various amplitudes (−50 to +50 in steps of 10 pA) and the corresponding local steady-state voltage response was recorded to compute *V*–*I* relationship. The slope of the linear fit to *V*–*I* curve formed the input resistance.

The root mean squared error (RMSE) between different response frequency profiles was computed as follows:

(22) $$\begin{array}{l} RMSE=\sqrt{\frac{1}{NK}\left(\overset{N}{\underset{SF=1}{\sum }}\overset{K}{\underset{Tr=1}{\sum }}{\left(NewRF_{SF}^{Tr}-BaseRF_{SF}^{Tr}\right)}^{2}\right)} \end{array}$$

where *N* is the total number of stimulus frequencies (1–50 Hz in steps of 1 Hz), *K* (=10) is the number of trials for each stimulus frequency, New-RF is the new response frequency and Base-RF is the baseline response frequency.

### Mutual information computation

Under the rate coding schema, mutual information, *I*_m_, between stimulus (different input frequencies) and output (response frequency) was computed as the difference between total response entropy, *H*, and noise entropy, *H*_noise_, (Dayan and Abbott, 2001):

(23) $$\begin{array}{l} I_{m}=H-H_{\text{noise}} \end{array}$$

Total response entropy, *H*, was computed as:

(24) $$\begin{array}{l} H=-\underset{r}{\sum }p\left[r\right]log_{2}\left(p\left[r\right]\right) \end{array}$$

where *p*[r] is the response probability distribution of response frequency, *r*, computed over the range of various stimulus frequencies, *s*:

(25) $$\begin{array}{l} p\left[r\right]=\underset{s}{\sum }p\left[\text{r }|\text{s}\right]\;p\left[s\right] \end{array}$$

A given input stimulus frequency, *s*, fed to various synapses was varied between 1–50 Hz in steps of 1 Hz. Each stimulus frequency was represented 10 times and input timings across all synapses for a given stimulus frequency were taken from independent Poisson distribution, thus, providing variability in output response frequencies across 10 trials. From this variability in output response frequencies across 10 trials, mean and standard deviation in response frequencies were computed and used to generate normalized Gaussian distribution, thus, providing us with *p*[r|s]. This procedure was repeated for each stimulus frequency. For computing *p*[s], the distribution of the applied stimulus frequencies is uniform; Each distinct stimulus frequency was presented the same number of times, so the probability of presenting any given stimulus frequency is equally likely. To compute the noise entropy, the first entropy of response for a given stimulus frequency was computed as:

(26) $$\begin{array}{l} Hs=-\underset{r}{\sum }p\left[\text{r}|\text{s}\right]\text{l}o{\text{g}}_{2}\left(p\left[\text{r}|\text{s}\right]\right) \end{array}$$

then noise entropy was computed as:

(27) $$\begin{array}{l} H_{noise}=\underset{s}{\sum }p\left[s\right]H_{s} \end{array}$$

### Generation of place field

One hundred fifty-one synapses (base theta synapses) were placed on the apical side of the dendritic arbor, distributed over the range of 12.5–286.7 μm away from the soma, to generate baseline theta oscillations. Additional 61 synapses (place theta synapse), distributed over a similar distance range as that of base theta synapses, were placed to induce place field activity. Base theta synapses were modeled using only AMPA receptor-like conductance whereas place theta synapses were modeled as co-localization of AMPA and NMDA receptor currents. Only place theta synapses undergo calcium-dependent synaptic plasticity. This pattern of using two distinct sources of theta activity was motivated by the fact that intracellular theta oscillation amplitude, and hence theta power, is significantly higher during place field compared to a non-place field activity, whereas theta oscillation frequency did not change significantly (Harvey et al., 2009). Moreover, to account for the asymmetric ramping depolarization (Harvey et al., 2009), we injected asymmetric ramping current at the soma during place field activity. Peak depolarization obtained during ramping current injection was around 4 mV (Harvey et al., 2009).

Stimulation timings of base theta and place theta synapses were Gaussian-modulated with a standard deviation set at one-eight of the 8 Hz oscillatory cycle (Sinha and Narayanan, 2015). The number of stimulating inputs to a synapse was governed by the distribution (Schomburg et al., 2012; Sinha and Narayanan, 2015):

(28) $$\begin{array}{l} N\left(t\right)=Aexp\left(-\frac{{\left(mod\left(t,T_{\theta }\right)-T_{\theta }/2\right)}^{2}}{2\sigma^{2}}\right) \end{array}$$

where *T*_θ_ represents the time period of the theta oscillations (125 ms for 8 Hz), σ = *T*_θ_/8, mod represents the modulo function, and *A* is the scaling factor and was set to unity.

With this kind of distribution and stimulation pattern of base theta and place theta synapses, the simulation was run for 10 s. Base theta synapses were stimulated throughout this time window, whereas place theta synapses were stimulated from 3–8 s, and correspondingly asymmetric depolarization ramp current was delivered at soma within this time window of 3–8 s. Spike timings of action potentials fired during 10 s were convolved with a normalized Gaussian window of 300 ms standard deviation to obtain a smooth place field profile.

### Computational details

All simulations were performed using NEURON simulation environment (Carnevale and Hines, 2006). For all simulations, the temperature was set at 34°C and ion-channels kinetics was appropriately adjusted based upon experimentally determined q10 factors. The integration time constant, for solving various differential equations, was set to be 25 μs. Simulations involving Poisson-modulated synaptic stimulation (Figures 1–5) were run at −65 mV, whereas simulations involving Gaussian-modulated synaptic stimulation (Figures 6, 7) were run at −66 mV. Data analyses involving computation of root mean squared error, mutual information, and smooth place field generation were done using custom-built software written within IGOR Pro (Wavemetrics).

## Results

Experimental evidence suggest that induction of bidirectional synaptic plasticity is accompanied by bidirectional changes in HCN conductance (Fan et al., 2005; Brager and Johnston, 2007; Narayanan and Johnston, 2007; Campanac et al., 2008). This bidirectional plasticity in HCN conductance has been postulated as a key regulator of input/output response homeostasis and information transfer (van Welie et al., 2004; Brager and Johnston, 2007; Narayanan and Johnston, 2007, 2010). Moreover, a previous computational framework has suggested a linear relationship between synaptic plasticity and HCN conductance plasticity for maintaining input/output response homeostasis and robust mutual information transfer (Honnuraiah and Narayanan, 2013). In this study, we examined whether the previously derived linear relationship between synaptic plasticity and HCN conductance plasticity for maintaining input/output response homeostasis and robust information transfer could be extended to a multi-compartmental model having multiple synapses and gradients of various ion-channels. This question is particularly important, especially given the fact that previous computational frameworks employed a single compartmental model, devoid of gradients in ion channels, and having a single synapse (Honnuraiah and Narayanan, 2013).

### Linear relationship between synaptic plasticity and HCN conductance plasticity maintains homeostasis of input/output response profile

We employed 3D reconstructed morphology of hippocampal CA1 pyramidal neuron (Figure 1A), which expressed five different voltage-gated ion channels (VGICs): Na^+^, *A*-type K^+^ (KA), delayed rectifier K^+^ (KDR), *T*-type Ca^++^ (CaT), and hyperpolarization-activated *h* (HCN) channels (Figure 1B), as described previously (Rathour and Narayanan, 2014; Rathour and Kaphzan, 2022) (also see Materials and methods). With this pattern of distribution of VGICs (Figure 1B), the model neuron was able to satisfy various experimental constrains regarding the co-existence of six functional maps along the same neuronal topography (Rathour and Narayanan, 2014). We placed 303 synapses on the apical side of the dendritic arbor (green region in Figure 1A) and tuned the permeability values of these synapses so that somatic EPSP amplitudes of all synapses were highly similar to each other (Figure 1C), therefore, maintaining dendritic democracy (Hausser, 2001). Toward this end, we had a model neuron expressing gradients of various VGICs and AMPA receptor conductance (Figures 1B, C). Next, we assessed the synaptically driven input/output response profile of this model neuron by stimulating all synapses at given frequencies (1–50 Hz in steps of 1 Hz) 10 times (Figure 1D). For each trial, stimulating timings of all synapses were drawn from independent Poisson distribution, thus, providing a response variability across trials for the given stimulus frequencies. To this end, we had only AMPA receptor type conductance at the synapses, given that baseline synaptic transmission is largely carried by AMPA receptors. Next, we examined whether induction/expression of synaptic plasticity disrupts these response profiles (Figure 1D) and if so, could they be restored by HCN conductance plasticity using previously derived linear relationship between synaptic plasticity and HCN conductance plasticity for maintaining input/output response homeostasis.

To answer this question, we introduced NMDA receptors at synapses along with AMPA receptors. Permeability values of NMDA receptors were defined by NMDA-to-AMPA ratio (NAR) for any given synapse, which was set to be 1.5 for all synapses. To induce synaptic plasticity, individual synapses were assigned a stimulus frequency by random sampling from the uniform distribution in the range of 4–12 Hz. All synapses were stimulated simultaneously and stimulating timings of synapses were determined by independent Poisson distributions. Owing to synaptic stimulation, and consequent entry of Ca^++^ from NMDA receptors and *T*-type Ca^++^ channels, synaptic weights evolved (Figure 1F) based upon intracellular Ca^++^ levels (Figure 1E). At the end of the simulation most synapses expressed LTP whereas few synapses underwent LTD (Figure 1G). When assessed for correlation between final synaptic weights and stimulus frequencies or final synaptic weights and location of synapses, we found weak correlations; *R*^2^ = 0.046 for final synaptic weight and stimulus frequency, and *R*^2^ = 0.326 for final synaptic weight and location of the synapse. These results suggest that synaptic plasticity was not just a function of either stimulus frequency or AMPA and NMDA receptor permeability values. This result should be expected given that the synaptic plasticity response profile is determined by multiple factors including synaptic and active membrane properties. Consequent to the increase in synaptic weights, somatic EPSP amplitude also increased (Figure 1H). Next, we assessed the synaptically driven input/output response profiles after the expression of synaptic plasticity. We found that after the expression of synaptic plasticity, input/output response profiles shifted toward the left (Figure 1I) owing to the increase in the synaptic drive following synaptic plasticity (Figure 1H). This shift in input/output response profiles constitutes a perturbation for a given input pattern, and requires some kind of homeostatic mechanism to restore the input/output response profile (Honnuraiah and Narayanan, 2013).

Plasticity in HCN conductance along with synaptic plasticity has been postulated to play a key role in maintaining homeostasis of the input/output response profile (van Welie et al., 2004; Fan et al., 2005; Brager and Johnston, 2007; Narayanan and Johnston, 2007, 2010). A quantitative modeling study showed that a linear relationship between synaptic plasticity and HCN conductance plasticity is sufficient for maintaining the homeostasis of the input/output response profile (Honnuraiah and Narayanan, 2013). Hence, we employed this linear relationship (Figure 2A), derived from a single-compartment model having a single synapse (Honnuraiah and Narayanan, 2013), to our morphologically realistic model that has multiple synapses and gradients of various VGICs. We found that increasing the slope of the linear relationship between synaptic plasticity and HCN conductance plasticity shifted the input/output response profiles toward the right (Figure 2B). Next, we employed root mean squared error (RMSE) as a measure to achieve homeostasis of input/output response profile. RMSE between the baseline response frequency profile and the response frequency profile obtained after synaptic and HCN conductance plasticity yielded inverted bell shaped curve as a function of the slope of the linear relationship (Figure 2C). Slope at which RMSE reached minimum value was taken as the optimal slope for yielding the homeostasis of input/output response profile. Looking at the evolution of synaptic weights during concurrent synaptic and HCN conductance plasticity (Figure 2D), we found that the overall change in magnitude of synaptic weights was lesser compared to the one achieved with only synaptic plasticity due to synaptic stimulation and Ca^++^ influx (compare Figures 1F vs. 2D). This is due to the concurrent increase in HCN conductance (Figure 2E). This decrease in magnitude of synaptic plasticity is also reflected in distribution of final synaptic weights (Figure 2F), thereby leading to decreased somatic EPSP amplitudes (Figure 2G). Owing to increase in HCN conductance during synaptic and HCN conductance plasticity (Figure 2E), input resistance (Figure 2H) and intrinsic firing rate (Figure 2I) decreased as observed experimentally (Fan et al., 2005; Narayanan and Johnston, 2007). Taken together, the herein results suggest that a linear relationship between synaptic plasticity and HCN conductance plasticity is sufficient for maintaining the homeostasis of input/output response profiles in a multi compartmental neuronal model having various synapses and gradients of ion channels.

### Optimal slope of the linear relationship decreases with increase in baseline synaptic permeability values and HCN conductance

Given that synaptic gradient along the neuronal arbor can undergo scaling depending upon the incoming network activity (Turrigiano, 1999; Turrigiano and Nelson, 2004), we asked whether scaling of the synaptic gradient in our model would change the optimal slope of the linear relationship between synaptic plasticity and HCN conductance plasticity for maintaining the homeostasis of input/output response profile. To test this, we multiplied the baseline synaptic permeability values of our base model (Figure 1C) by various scaling factors (Supplementary Figure S1A) to get the unitary EPSP amplitude of different magnitudes (Supplementary Figure S1B), while making sure that dendritic democracy is maintained. First, we generated synaptically driven input/output response profiles under baseline conditions for different scaling factors (black traces in Supplementary Figures S1C–F). We found that an increase in scaling factor shifted the baseline input/output response profiles toward the left (compare black traces in Supplementary Figures S1C–F; right), owing to the increase in synaptic drive. Next, we induced synaptic plasticity in different models having different scaling factors by employing the aforementioned protocol and assessed their input/output response profiles after the expression of synaptic plasticity. We found that expression of synaptic plasticity resulted in a further leftward shift in input/output response profiles for various scaling factors (red traces in Supplementary Figures S1C–F; right), and that the input/output response profile with the highest scaling factor exhibited a drop for high stimulus frequencies (red trace in Supplementary Figure S1F; right), owing to depolarization-induced inactivation of sodium channels.

Next, we introduced HCN conductance plasticity along with synaptic plasticity with different slopes of the linear relationship (Figure 2A) for various scaling factors. As found previously (Figure 2C), RMSE between the baseline response frequency profile and the response frequency profile obtained after synaptic and HCN conductance plasticity yielded an inverted bell-shaped curve as a function of the slope of the linear relationship (Supplementary Figures S1C–F; left). Looking at the optimal slope that produces homeostasis of input/output response profiles, we found that the optimal slope decreases as the scaling factor increases (Supplementary Figures S1C–F; left, Supplementary Figure S1G). We also found that irrespective of the scaling factor, baseline input/output response profiles exhibited type-I firing rate profiles, whereas after homeostasis of input/output response profiles, the firing rate profiles became type-II through an increase in HCN conductance (compare black and green traces Supplementary Figures S1C–F; right) (Connor, 1975; Drion et al., 2015).

Since it is established that the expression of HCN conductance is highly variable within the homogenous neuronal population, and synaptic plasticity protocols induce variable change in the magnitude of HCN conductance (Fan et al., 2005; Narayanan and Johnston, 2007; Campanac et al., 2008), we examined the dependence of the optimal slope for maintaining the homeostasis of input/output response profile against baseline HCN conductance. To test this, we employed a model neuron with the highest scaling factor (Supplementary Figure S1F; right). The reason for choosing this model as opposed to the baseline model (Figure 1C) was to maintain a significant synaptic drive for providing input/output response profiles with various baseline HCN conductance values. We varied the baseline HCN conductance values and generated distinct gradients of HCN conductance (Supplementary Figure S2A). Thereafter, we generated synaptically driven input/output response profiles under baseline conditions for the different baseline HCN conductance values (black traces in Supplementary Figures S2B–F; left). We found that increasing the baseline HCN conductance values shifted baseline input/output response profiles toward the right (compare black traces in Supplementary Figures S2B–F; left), owing to a decrease in intrinsic excitability. Next, we induced synaptic plasticity in different models having different HCN conductance gradients and assessed their input/output response profiles after the expression of synaptic plasticity. We found that expression of synaptic plasticity resulted in a leftward shift and an increase in input/output response frequencies for various baseline HCN conductance values (red traces in Supplementary Figures S2B–F; left). In all cases, the input/output response profile exhibited a drop for high stimulus frequencies (red trace in Supplementary Figures S2B–F; left), owing to depolarization-induced inactivation of sodium channels.

Next, we introduced HCN conductance plasticity along with synaptic plasticity with different slopes of the linear relationship for various base HCN conductance values. As previously, by employing RMSE as a measure to obtain optimal slope (Figure 2C), we found that the optimal slope decreased with the increase in baseline HCN conductance values (Supplementary Figures S2B–F; left, Supplementary Figure S2H). Here also, we observed the switch in input/output response profiles from type-I to type-II after achieving input/output response homeostasis for various baseline HCN conductance values (compare black and green traces Supplementary Figures S2B–F; left). Taken together, these results suggest that the linear relationship between synaptic and HCN conductance plasticity could be employed for a wide range of synaptic permeability and HCN conductance values in order to maintain homeostasis of input/output response profiles, where an optimal slope of the linear relationship is critically dependent upon baseline synaptic permeability values and HCN conductance levels.

### HCN conductance plasticity together with synaptic plasticity maintains homeostasis of input/output response profile during repeated synaptic stimulation

The major negative impact of Hebbian plasticity is the positive feedback loop incurred by an increase/decrease in AMPA and/or NMDA receptor permeability during repetitive synaptic stimulation. These positive feedback loops could result in cessation of action potential firing either through a reduction in synaptic drive during LTD or enhanced synaptic drive during LTP, which eventually could lead to depolarization-induced inactivation of sodium channels. Therefore, it is essential to address the validity of a linear relationship between synaptic and HCN conductance plasticity in maintaining the homeostasis of input/output response profiles during repetitive synaptic stimulation. To test this, we employed our baseline model (Figure 1C) and examined whether a linear relationship between synaptic and HCN conductance plasticity is sufficient for maintaining homeostasis of input/output response profiles during repetitive synaptic stimulation. We induced synaptic plasticity by stimulating individual synapses at a given frequency, drawn from the uniform distribution of 4–12 Hz range, where stimulating timings of each synapse were determined by independent Poisson distribution. After the expression of synaptic plasticity, permeability values of AMPA receptors for each synapse were updated according to the final synaptic weight and then the model neuron was assessed for synaptically driven input/output response profile. During the next synaptic stimulation, synapses expressed updated AMPA receptor permeability values while the spatio-temporal synaptic stimulation pattern was kept constant. This procedure was repeated seven times. Increasing evidence suggest that along with AMPA receptor conductance, NMDA receptor conductance could also undergo plasticity (Hunt and Castillo, 2012), and in certain cases it is found that plasticity in NMDA receptor conductance along with AMPA receptor conductance maintains NMDA-to-AMPA ratio constant before and after synaptic plasticity (Watt et al., 2004). Therefore, in our simulations, following the change in AMPA receptor expression due to plasticity we updated NMDA receptor permeability values so that the NMDA-to-AMPA ratio was kept at 1.5 throughout all iterations of synaptic stimulation. This means that after every iteration, AMPA and NMDA receptor conductance expressed synaptic plasticity. Therefore, our model successfully incorporated positive feedback loops associated with both AMPA and NMDA receptors.

First, we tested the effect of repetitive synaptic plasticity on input/output response profiles under the condition of only synaptic plasticity. We found that input/output response profiles of the model neuron kept on shifting toward the left during repetitive synaptic stimulation (Figure 3A), owing to the increased synaptic drive after the expression of LTP (Figure 1G). However, after certain iterations, the model neuron ceased firing at high stimulation frequencies and eventually stopped firing for any stimulation frequency owing to depolarization-induced inactivation of sodium channels due to increase in synaptic drive during repetitive synaptic stimulation (Figure 3A). This increase in response frequency followed by cessation of firing of action potential during repetitive synaptic stimulation was also reflected in the RMSE for various iterations (Figure 3B). The RMSE exhibited an initial increase as the response frequency increased and later on it decreased as the model neuron underwent depolarization-induced inactivation of sodium channels (Figure 3B). Given that during repetitive synaptic stimulation the model neuron completely lost input/output response homeostasis (Figure 3A), and given the fact that the loss of firing efficiency due to depolarization-induced inactivation of sodium channels could severely limit information transfer efficacy of neurons (Honnuraiah and Narayanan, 2013), we aimed to examine whether the linear relationship between synaptic plasticity and HCN conductance plasticity would be sufficient for maintaining homeostasis of input/output response profile during repetitive synaptic stimulation.

Our previous sensitivity analyses showed that the optimal slope of the linear relationship between synaptic and HCN conductance plasticity for maintaining input/output response homeostasis strongly depends on baseline synaptic permeability values and HCN conductance levels (Supplementary Figures S1, S2). Since synaptic permeability values and thereby HCN conductance levels alter after each iteration of synaptic stimulation, the optimal slope of the linear relationship for maintaining homeostasis of the input/output response profile change for each iteration of synaptic stimulation. By employing RMSE as a measure to obtain the optimal slope (Figure 2C), we found that the optimal slope for reducing RMSE decreased as a function of iteration number (Figure 3C). This is expected given that overall synaptic permeability value and thereby HCN conductance magnitude increase after every iteration of synaptic stimulation, hence, the optimal slope would decrease accordingly (Figures 3, 4). We also found that for each iteration of synaptic stimulation, RMSE was lesser (Figure 3D green line) as compared to only synaptic plasticity condition (Figure 3D red line) and it did not change much with an increase in iteration number of synaptic stimulation (Figure 3D). Finally, after looking at the input/output response profiles for each iteration of synaptic stimulation, under the condition of synaptic and HCN conductance plasticity, we found that homeostasis of input/output response profiles was maintained throughout various iterations of synaptic stimulation (Figure 3E). These results suggest that a linear relationship between synaptic and HCN conductance plasticity is sufficient for maintaining homeostasis of input/output response profiles during repetitive synaptic stimulations, where the slope of the linear relationship is determined by synaptic permeability values and HCN conductance levels.

Although, this analysis confirms that the linear relationship between synaptic and HCN conductance plasticity is sufficient for maintaining homeostasis of input/output response profiles during repetitive synaptic stimulation, it is still unclear whether this homeostasis of input/output response profiles is also accompanied by homeostasis of response frequency distribution. This is particularly important, especially given the fact that response frequency distribution could profoundly affect the information encoding capabilities of a neuron (Stemmler and Koch, 1999; Triesch, 2007). Therefore, we looked into the distribution of response frequencies. We found that under the baseline condition the distribution of response frequencies inclined to be uniform (Figure 3F), whereas after the first iteration of introducing only synaptic plasticity, response frequency distribution shifted toward exponential (Figure 3G). On the other hand, under the condition of introducing both synaptic and HCN conductance plasticity, distribution response frequency shifted toward bimodal after the first iteration (Figure 3H), which eventually converged to be lognormal toward the last iteration (Figure 3I). This result suggests that homeostasis of input/output response profiles could be achieved with different distributions of response frequencies.

### Homeostasis of input/output response profiles occurs at the expense of robust mutual information transfer

Our previous results suggest that a linear relationship between synaptic and HCN conductance plasticity is sufficient for maintaining homeostasis of input/output response profiles during repetitive synaptic stimulation (Figure 3), however, it is not clear whether homeostasis of input/output response profiles is accompanied by maintenance of robust information transfer. The general notion is that the homeostasis of input/output response profiles is sufficient for maintaining robust information transfer (Triesch, 2007; Honnuraiah and Narayanan, 2013). Therefore, we analyzed our data under rate coding schema to test whether the homeostasis of the input/output response profile was accompanied by robust information transfer.

At first, we looked at the probability distributions of response frequencies for various stimulus frequencies. We observed that probability distributions of response frequencies for various stimulus frequencies were confined within the range of 0–45 Hz under baseline conditions (Figure 4A), whereas during the introduction of only synaptic plasticity, these distributions shifted toward the right in the first iteration of synaptic stimulation (Figure 4B) owing to increase in synaptic drive. On the other hand, distributions of response frequency for various stimulus frequencies after the 7th iteration of synaptic stimulation converged toward lowermost values with distributions heavily overlapping with each other (Figure 4C). This is expected, given that after the 7th iteration of synaptic stimulation, the model neuron ceased firing owing to depolarization-induced inactivation of sodium channels (Figure 3A). Next, we looked into the probability distributions of response frequencies for various stimulus frequencies under the condition of synaptic and HCN conductance plasticity. We found that the probability distributions of response frequencies were maintained within similar ranges as to that of the baseline condition (Figure 4A) after the first iteration of synaptic stimulation (Figure 4D). Analyzing the distributions of response frequencies after the 7th iteration of synaptic stimulation, we found that the overall distribution shifted toward the right, but still maintained the large range of response frequencies (Figure 4E). We also noticed that the overall variability in response frequencies was increased after the 7th iteration of synaptic stimulation, which caused the distributions of response frequencies across various stimulus frequencies to strongly overlap (Figure 4E). This result suggests that although a linear relationship between synaptic and HCN conductance plasticity could maintain a similar range of response frequencies, it could not account for the shape of individual probability distributions of response frequencies across various stimulus frequencies.

Looking at the distributions of response probability (derived from traces shown in Figures 4A–E) under various conditions, we found that for only synaptic plasticity condition, the response probability distribution shifted toward the right compared to baseline condition for the first iteration of synaptic stimulation, while after the 7th iteration of synaptic stimulation the response probabilities converged toward the lowermost values (Figure 4F). On the other hand, the distribution of response probability, under the condition of synaptic and HCN conductance plasticity, was spread over the range of response frequencies irrespective of the iteration of synaptic stimulation (Figure 4G). Turning toward the mutual information, we found that irrespective of the condition, whether only synaptic plasticity or synaptic and HCN conductance plasticity, mutual information decreased as a function of iteration number (Figure 4H). We also noticed that mutual information, under the condition of only synaptic plasticity, was higher compared to the condition of synaptic and HCN conductance plasticity for the initial few iterations of synaptic stimulation, while after that mutual information was lower (Figure 4H). This result suggests that homeostasis of input/output response profiles is not sufficient in maintaining robust information transfer during repetitive synaptic stimulation.

### Optimal slope for information maximization does not rescue the decrease in mutual information during repetitive synaptic stimulation

Our previous results showed that homeostasis of input/output response profiles was accompanied by a decrease in mutual information transfer (Figures 3, 4). Next, we investigated whether this loss of mutual information transfer could be rescued by optimizing the slope of the linear relationship between synaptic and HCN conductance plasticity, in order to maximize the information transfer during repetitive synaptic stimulation. In our previous analysis we computed mutual information in 0–50 Hz stimulus frequency range. But given that our model neuron does not fire action potentials until 10 Hz (Figure 1D), we computed mutual information in 10–50 Hz stimulus frequency range to maximize mutual information. While doing this, we computed mutual information for various slopes of linear relationships between synaptic and HCN conductance plasticity; and the slope, where mutual information exhibited maximum value, was taken as the optimal slope (Figure 5A). We found that the optimal slope decreased with the increase in the number of iterations of synaptic stimulations (Figure 5B). Looking into the mutual information as a function of iteration number and for the corresponding optimal slope, we found that mutual information still decreased with the increase in iteration of synaptic stimulation (Figure 5C) [mutual information (bits); 3.96 for baseline; 3.74 for 1st iteration; 2.49 for 7th iteration]. Correspondingly, looking at the input/output response profiles, we found that the homeostasis of input/output response profiles was completely lost (Figure 5D), and RMSE between baseline response profile and response profile obtained after synaptic and HCN conductance plasticity increased as a function of number of iteration (Figure 5E). Compared to aforementioned results for maintaining the homeostasis of input/output response profiles during repeated synaptic stimulation (Figure 3), where the distribution of response frequencies became bimodal and lognormal after 1st and 7th synaptic stimulation, respectively (Figures 3H, I), here, we found that response frequencies retained the exponential distribution after the 1st and 7th synaptic stimulations (Figure 5F). These results suggest that linear relationship between synaptic and HCN conductance plasticity is sufficient for maintaining the homeostasis of input/output response profiles (Figure 3) but could not sustain robust information transfer irrespective of the optimal slope for RMSE minimization or mutual information maximization (Figures 4, 5).

To understand the mechanistic basis for the reduction in information transfer for the two above-mentioned cases, we looked into changes in response entropy as well as noise entropy with repeated synaptic stimulation. Usually, mutual information can be reduced by either reduction in response entropy, or increase in noise entropy, or by a combination of the two (Equation 23). Hence, we computed the percentage change in response and noise entropy with respect to baseline values as a function of the iteration number of synaptic stimulation. We found that under the scenario of RMSE minimization, response entropy did not change significantly whereas noise entropy increased up to three-fold by the end of the 3rd iteration of synaptic stimulation and later on stabilized (Figure 5G). On the other hand, under the condition of mutual information maximization, noise entropy did not change much by the end of the 3rd iteration of synaptic stimulation, but afterward, it increased while response entropy did not change significantly throughout the various iterations of synaptic stimulation (Figure 5H). These results suggest that irrespective of the condition of slope optimization, the reduction in mutual information is due to the increase in noise entropy.

### Synaptic plasticity disrupts place field activity in the model neuron

So far, our study focused on Poisson-distributed synaptic stimulation in order to understand the relationship between synaptic and HCN conductance plasticity in enabling homeostasis of input/output response profiles and its consequence on mutual information transfer. Although Poisson-distributed synaptic inputs have been widely used in computational models for understanding various aspects of neuronal and/or network functioning, it is noteworthy that these inputs were shown to occur largely in cortical regions (Softky and Koch, 1993; Compte et al., 2003), whereas hippocampal neurons are driven by Gaussian modulated synaptic inputs during exploratory behavior and rapid eye moment (REM) sleep (Buzsaki, 2002). Therefore, it is important to validate the usefulness of a linear relationship between synaptic and HCN conductance plasticity for enabling homeostasis of input/output response profiles under the condition of Gaussian-modulated synaptic inputs. To do this, we employed a place cell model given that place field activity could be regulated by both synaptic plasticity as well as HCN conductance (Mehta et al., 2000; Hussaini et al., 2011).

To generate place field activity, first, we placed 151 synapses (base theta synapses) on the apical side of the dendritic arbor (Figure 6A) and activated them using Gaussian-modulated input timings (Schomburg et al., 2012; Sinha and Narayanan, 2015) to obtain baseline theta frequency membrane potential oscillations at around 8 Hz with sparse firing of action potentials (Figure 6B; non-shaded region). Then, we introduced another 61 synapses (place theta synapses), along the same dendritic arbor to that of base theta synapses, and activated them to induce place field activity (Figure 6B; shaded region). With this kind of synapse distribution and activation pattern, we ran the simulation for 10 s. Base theta synapses were activated throughout this 10 s period, whereas place theta synapses were activated between 3–8 s period (Figure 6B; shaded region). Correspondingly, an asymmetric depolarizing ramp current was delivered at the soma during 3–8 s period. With this kind of setup, our model place field activity was able to satisfy various experimental observations; (1) During non-place field activity, action potential firing was sparse (Harvey et al., 2009). (2) Power of intracellular theta membrane potential oscillations was higher during place field activity compared to non-place field activity. (3) Average membrane potential was almost 4 mV depolarized during place field activity compared to non-place field activity.

Next, we introduced synaptic plasticity during place field activity. Given that theta frequency synaptic stimulation is sufficient for inducing synaptic plasticity *in vitro*, we examined whether Gaussian-modulated synaptic inputs at theta frequency are sufficient for inducing synaptic plasticity in our place field model. Therefore, we assessed the evolution of synaptic weights of place theta synapses during place field activity, given that only place theta synapses were allowed to undergo synaptic plasticity. We found that during the ongoing theta activity, the weights of place theta synapses evolved during the place field activity (Figure 6C), and a number of place theta synapses exhibited robust LTP at the end of that place field activity (Figure 6D). Correspondingly, unitary EPSP amplitudes at soma also increased after the expression of LTP (Figure 6E). Looking at the membrane potential dynamics during place field activity during which synaptic weights also evolved (Figures 6C, D), we found that as synaptic weights evolved during place field activity, the firing rate also increased and eventually, synaptic drive increased so much that depolarization-induced inactivation of sodium channels kicked in and the model neuron ceased to fire action potentials during place field activity (Figures 6F, G). This cessation of action potential firing constitutes a loss of information and has to be rescued in order to maintain place field activity.

### Linear relationship between synaptic and HCN conductance plasticity is sufficient for maintaining stable place field activity

To test whether a linear relationship between synaptic and HCN conductance plasticity is sufficient for maintaining stable place field activity, we introduced HCN conductance plasticity along with synaptic plasticity using a linear relationship (Figure 7A) as described previously (Honnuraiah and Narayanan, 2013). We employed RMSE between baseline place field profile and place field profile obtained after synaptic and HCN conductance plasticity as a measure of stability of place field activity. We found that introducing HCN conductance plasticity along with synaptic plasticity, using different slopes, resulted in the restoration of place field activity (Figure 7B), where RMSE exhibited an inverted bell-shaped form as the function of the slope of that linear relationship (Figure 7C). Looking at the membrane potential dynamics during the optimal slope of the linear relationship between synaptic and HCN conductance plasticity, we found that place field activity was completely restored (Figure 7D). Owing to the increase in HCN conductance during synaptic and HCN conductance plasticity (Figure 7E), the evolution of synaptic weights was decreased (Figures 7F, G) and correspondingly, somatic unitary EPSP amplitude (Figure 7H) and input resistance (Figure 7I) along the neuronal trunk also decreased. These results suggest that a linear relationship between synaptic and HCN conductance plasticity is sufficient for maintaining stable place field activity and also point that a linear relationship between synaptic and HCN conductance plasticity could be utilized for enabling homeostasis of input/output response profiles under the condition of Gaussian-modulated inputs.

## Discussion

Homeostatic regulation of neuronal physiological properties lies at the heart of normal brain functioning. Various homeostatic mechanisms at synaptic, intrinsic, and molecular level act either independently or in concert with each other to provide stability to various neurophysiological properties (Turrigiano and Nelson, 2000, 2004; Turrigiano, 2007, 2011). To this end, in this study, we show that the previously derived linear relationship between synaptic plasticity and HCN conductance plasticity in a single compartmental model having a single synapse for maintaining input/output response homeostasis (Honnuraiah and Narayanan, 2013) could be extended further to a multi-compartmental model having multiple synapses and gradients of various ion-channels (Figures 1, 2), where the optimal slope of the linear relationship between synaptic and HCN conductance plasticity is heavily dependent upon synaptic permeability values and base HCN conductance levels. We also found that various distributions of response frequencies could yield similar input/output response profiles (Figure 3). Therefore, we further show that homeostasis of input/output response profiles does not necessarily translate to robust information transfer (Figures 4, 5), given that information transfer heavily depends upon the distribution of response frequencies (Stemmler and Koch, 1999; Triesch, 2007). Lastly, using a Gaussian-modulated input pattern, we show that HCN conductance plasticity along with synaptic plasticity could provide stability to place field activity (Figures 6, 7).

### Homeostasis of neuronal properties and various ion channels

Homeostasis of neuronal properties is extremely important for physiological, behavioral, and cognitive functions. Here, we specifically focused on the homeostasis of input/output response profiles during synaptic plasticity. Using biophysically rooted and experimentally constrained computational principles, we show that a linear relationship between synaptic plasticity and HCN conductance plasticity is sufficient for maintaining input/output response homeostasis. Although, homeostasis of input/output response profiles was achieved by concurrent synaptic and HCN conductance plasticity, certain intrinsic physiological properties that are mediated/regulated by HCN conductance, exhibited huge change, commonly not observed in typical plasticity experiments. For example, in our model, the change in somatic input resistance after HCN conductance plasticity was very high compared to typical experiments (Figure 2H). Several factors could contribute to this anomaly between modeling and experimental data. Prominent among these is the total synaptic weight change in our model that could be far greater than the one found in experiments, where plasticity is highly localized. In our model, a large number of synapses distributed over a wide range of the neuronal tree underwent LTP (Figure 2F). Therefore, in order to restore input/output response homeostasis, a sizable amount of change in HCN conductance was required. This rationale was corroborated by the results of homeostasis of place field activity during synaptic plasticity in our model. Specifically, place field activity was generated using a lesser number of synapses, as a result, the plasticity in HCN conductance for maintaining place field activity was lesser, and subsequently, the change in input resistance was within experimentally observed ranges (Figures 6, 7). Here, we exclusively considered only HCN conductance plasticity during synaptic plasticity, whereas experimentally it is known that several other voltage-gated ion channels change during synaptic plasticity-inducing protocols (Frick and Johnston, 2005; Magee and Johnston, 2005; Lin et al., 2008; Narayanan and Johnston, 2012; Rathour and Narayanan, 2019). Therefore, a future model should also account for the plasticity in various other ion channels during synaptic plasticity and should delineate specific roles of various ion channels in contributing toward homeostasis of input/output response profiles. This is extremely important, given that each type of ion channel has its own influence on a given physiological property, therefore, combinatory dynamics of various ion channels should be accounted while achieving homeostasis of input/output response profiles and also constraining various physiological properties.

### Homeostasis and information transfer

Within the framework of rate coding schema, it is inferred that homeostasis of input/output response profiles maintains robust information transfer (Triesch, 2007; Honnuraiah and Narayanan, 2013). Our results challenge this notion as we observed that homeostasis of input/output response profiles occurred at the expense of information coding capabilities (Figures 3, 4). Information transfer heavily depends upon the distribution of response frequencies (Stemmler and Koch, 1999; Triesch, 2007). Therefore, looking into the distributions of response frequencies, we found that disparate distributions of response frequencies could yield similar input/output response profiles (Figure 3). This implies that for similar input/output response profiles, information transfer could be very different, as similar input/output response profiles are coming from different distributions of response frequencies (Figures 3, 4). This is one aspect of the dependence of information transfer on the distribution of response frequencies, which we found need not be the rule of thumb. Specifically, when we optimized the slope of the linear relationship between synaptic and HCN conductance plasticity for maximizing information transfer, we found that even with similar distributions of response frequencies, the model neuron had distinct information transfer capabilities (Figure 5). After probing into the mechanistic basis of this anomaly we found that it is noise entropy (a measure of response variability of the system itself), which played a critical role in determining information coding capabilities (Figure 5). Under both scenarios, homeostasis of input/output response profile or maximizing information transfer, noise entropy was increased. Although, we did not explore the mechanistic basis for the alteration of noise entropy with repeated synaptic stimulation, under both scenarios, homeostasis and information maximization, only two things changed in the system, synaptic permeability values and HCN conductance levels. Therefore, future studies should focus on exploring the relative contributions of synaptic and HCN conductance plasticity in determining information coding capabilities. Moreover, given that several other voltage-gated ion channels exhibit plasticity during synaptic plasticity-inducing protocols (Frick and Johnston, 2005; Magee and Johnston, 2005; Lin et al., 2008; Narayanan and Johnston, 2012; Rathour and Narayanan, 2019), the role of these channels should also be investigated in determining information coding capabilities along with homeostasis of intrinsic properties and input/output response profiles.

### Regenerative events, synaptic plasticity and input/output relationship

Hippocampal CA1 neuron dendrites posses a myriad of VGICs (Johnston et al., 1996; Migliore and Shepherd, 2002; Lai and Jan, 2006). The presence of these VGICs transform these dendrites into a powerful computational machinery (Llinas, 1988; Marder, 1998; Hutcheon and Yarom, 2000; London and Hausser, 2005; Johnston and Narayanan, 2008; Remme et al., 2010; O'Donnell and Nolan, 2011). One aspect of this is their capability of generating regenerative events (e.g., dendritic Na^+^ spikes, Ca^++^ spike and NMDA spikes) during a strong stimulus. The role of these regenerative events in modulating synaptic plasticity and neuronal physiology is well-established. Although we did not explicitly analyzed the role of such regenerative events in our study, in our model, during the induction of synaptic plasticity the stimulus was not strong, which mostly rules out the possibility for generation of regenerative events during the induction period. On the other hand, during input/output relationship stimulus was strong enough to produce high frequency firing and hence generation of regenerative events cannot be neglected. How these regenerative events affect input/outout relationship is a question which should be targeted in future studies. Moreover, in this study we did not include NMDA receptors while computing input/output relationship. But given that the role of NMDA receptors in generating regenerative events is well established, we believe that future studies should also focus on NMDA receptors and their role in modulating input/output relationship.

## Data availability statement

Publicly available datasets were analyzed in this study. This data can be found at: http://neuromorpho.org/.

## Author contributions

RKR and HK designed the research, analyzed the data, and wrote the paper. RKR performed the research. HK supervision and funding acquisition. All authors contributed to the article and approved the submitted version.

## Supplementary material

Supplementary Figure S1 Optimal slope of the linear relationship between synaptic and HCN conductance plasticity decreases with increase in baseline synaptic permeability. **(A, B)** Synaptic permeability values after scaling with various permeability scaling factors **(A)** and corresponding somatic EPSP amplitudes **(B)** plotted as a function of synaptic locations. **(C–F)** *Left*; Input/output response profiles of model neuron under baseline condition (black) and after synaptic plasticity (red) for various permeability scaling factors. Note the increase in baseline response firing rate after increasing synaptic permeability values. *Middle*; Root mean squared error (RMSE) between baseline input/output response profile and response profile obtained after synaptic and HCN conductance plasticity (green trace) as a function of slope of the liner relationship for various permeability scaling factors. Red dot denotes RMSE between baseline input/output response profile and response profile obtained after only synaptic plasticity. Arrow indicates optimal slope. *Right*; Input/output response profiles of model neuron under baseline condition (black), after synaptic plasticity (red) and after synaptic and HCN conductance plasticity (green) for various permeability scaling factors. Data is presented as mean ± SD. **(G)** Optimal slope of the linear relationship between synaptic and HCN conductance plasticity plotted as a function of synaptic scaling factor and baseline HCN conductance.

Supplementary Figure S2 Optimal slope of the linear relationship between synaptic and HCN conductance plasticity decreases with increase in baseline HCN conductance. **(A)** Schematic representation of various HCN conductance gradients tested. **(B–F)** *Left*; Input/output response profiles of model neuron under baseline condition (black) and after synaptic plasticity (red) for various baseline HCN conductance values. *Right*; Root mean squared error (RMSE) between baseline input/output response profile and response profile obtained after synaptic and HCN conductance plasticity (green trace) as a function of slope of the liner relationship for various baseline HCN conductance. Red dot denotes RMSE between baseline input/output response profile and response profile obtained after only synaptic plasticity. Data is presented as mean ± SD. **(G)** RMSE as a function of various baseline HCN conductance values. **(H)** Optimal slope of the linear relationship between synaptic and HCN conductance plasticity plotted as a function of baseline HCN conductance values.
