# Supplementary figures and images for: Synergies between synaptic and HCN channel plasticity dictates firing rate homeostasis and mutual information transfer in hippocampal model neuron
Source: Front Cell Neurosci. 2023 Mar 20;17:1096823. doi: 10.3389/fncel.2023.1096823 (PMC10067771; doi:10.3389/fncel.2023.1096823)

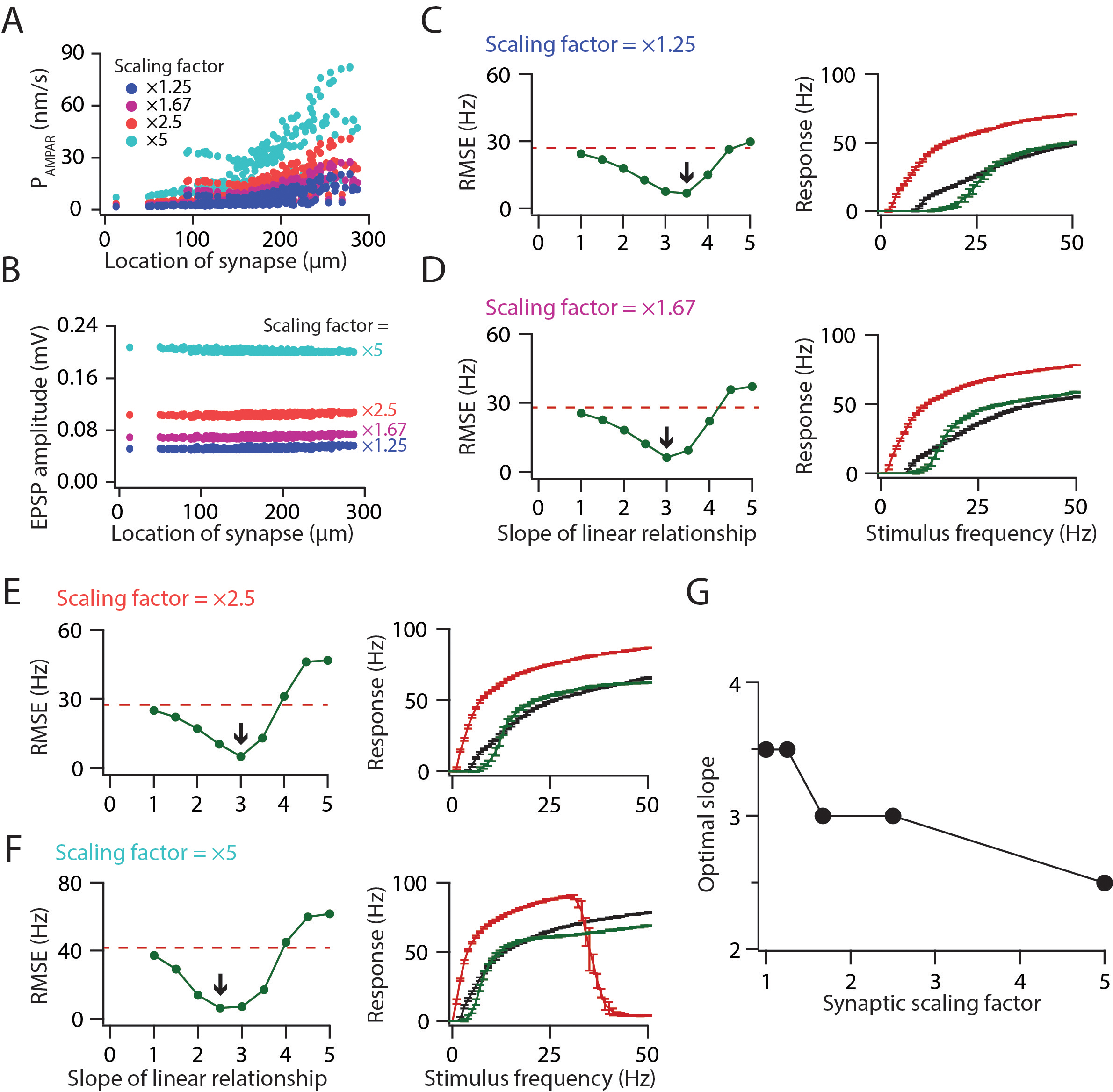

Supplement: Supplementary Figure S1 — Optimal slope of the linear relationship between synaptic and HCN conductance plasticity decreases with increase in baseline synaptic permeability. (A, B) Synaptic permeability values after scaling with various permeability scaling factors (A) and corresponding somatic EPSP amplitudes (B) plotted as a function of synaptic locations. (C–F) Left; Input/output response profiles of model neuron under baseline condition (black) and after synaptic plasticity (red) for various permeability scaling factors. Note the increase in baseline response firing rate after increasing synaptic permeability values. Middle; Root mean squared error (RMSE) between baseline input/output response profile and response profile obtained after synaptic and HCN conductance plasticity (green trace) as a function of slope of the liner relationship for various permeability scaling factors. Red dot denotes RMSE between baseline input/output response profile and response profile obtained after only synaptic plasticity. Arrow indicates optimal slope. Right; Input/output response profiles of model neuron under baseline condition (black), after synaptic plasticity (red) and after synaptic and HCN conductance plasticity (green) for various permeability scaling factors. Data is presented as mean ± SD. (G) Optimal slope of the linear relationship between synaptic and HCN conductance plasticity plotted as a function of synaptic scaling factor and baseline HCN conductance. [file Image_1.jpg]

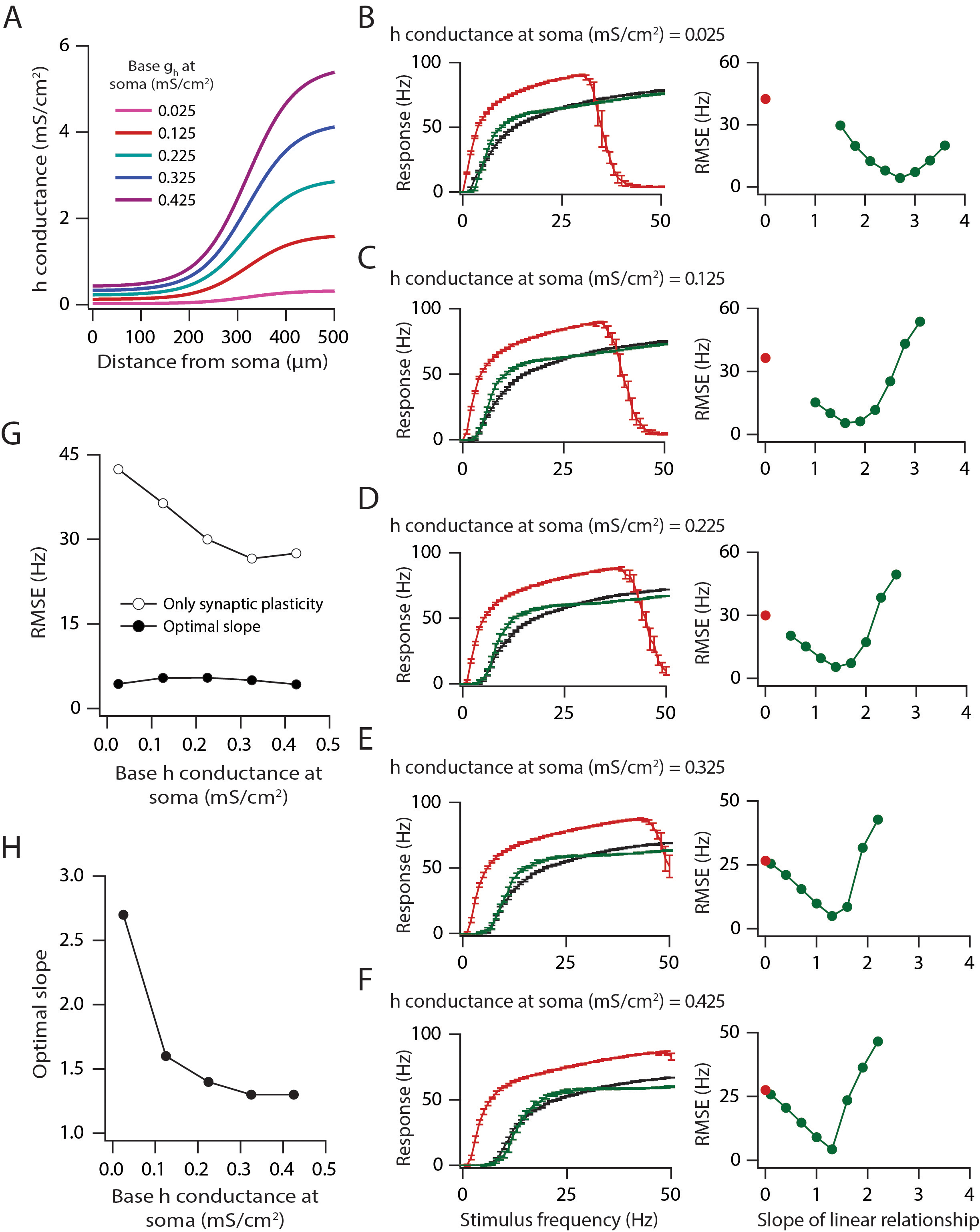

Supplement: Supplementary Figure S2 — Optimal slope of the linear relationship between synaptic and HCN conductance plasticity decreases with increase in baseline HCN conductance. (A) Schematic representation of various HCN conductance gradients tested. (B–F) Left; Input/output response profiles of model neuron under baseline condition (black) and after synaptic plasticity (red) for various baseline HCN conductance values. Right; Root mean squared error (RMSE) between baseline input/output response profile and response profile obtained after synaptic and HCN conductance plasticity (green trace) as a function of slope of the liner relationship for various baseline HCN conductance. Red dot denotes RMSE between baseline input/output response profile and response profile obtained after only synaptic plasticity. Data is presented as mean ± SD. (G) RMSE as a function of various baseline HCN conductance values. (H) Optimal slope of the linear relationship between synaptic and HCN conductance plasticity plotted as a function of baseline HCN conductance values. [file Image_2.jpg]
